# Essential role of zyxin in platelet biogenesis and glycoprotein Ib-IX surface expression

**DOI:** 10.1038/s41419-021-04246-x

**Published:** 2021-10-16

**Authors:** Rong Yan, Xinxin Ge, Ningbo Pang, Honglei Ye, Liuxia Yuan, Bin Cheng, Kangxi Zhou, Mengnan Yang, Yueyue Sun, Suqin Zhang, Zhongren Ding, Jincai Luo, Changgeng Ruan, Kesheng Dai

**Affiliations:** 1 Jiangsu Institute of Hematology, Cyrus Tang Medical Institute, The First Affiliated Hospital and Collaborative Innovation Center of Hematology, State Key Laboratory of Radiation Medicine and Protection, Soochow University, Key Laboratory of Thrombosis and Hemostasis, Ministry of Health, National Clinical Research Center for Hematological Diseases, Suzhou, 215006 China; 2 Department of Biochemistry and Molecular Biology, School of Basic Medical Sciences, Fudan University, Shanghai, 200032 China; 3 Laboratory of Vascular Biology, Institute of Molecular Medicine, Beijing Key Laboratory of Cardiometabolic Molecular Medicine, Peking University, Beijing, 100871 China

**Keywords:** Cytoskeleton, Disease genetics

## Abstract

Platelets are generated from the cytoplasm of megakaryocytes (MKs) via actin cytoskeleton reorganization. Zyxin is a focal adhesion protein and wildly expressed in eukaryotes to regulate actin remodeling. Zyxin is upregulated during megakaryocytic differentiation; however, the role of zyxin in thrombopoiesis is unknown. Here we show that zyxin ablation results in profound macrothrombocytopenia. Platelet lifespan and thrombopoietin level were comparable between wild-type and zyxin-deficient mice, but MK maturation, demarcation membrane system formation, and proplatelet generation were obviously impaired in the absence of zyxin. Differential proteomic analysis of proteins associated with macrothrombocytopenia revealed that glycoprotein (GP) Ib-IX was significantly reduced in zyxin-deficient platelets. Moreover, GPIb-IX surface level was decreased in zyxin-deficient MKs. Knockdown of zyxin in a human megakaryocytic cell line resulted in GPIbα degradation by lysosomes leading to the reduction of GPIb-IX surface level. We further found that zyxin was colocalized with vasodilator-stimulated phosphoprotein (VASP), and loss of zyxin caused diffuse distribution of VASP and actin cytoskeleton disorganization in both platelets and MKs. Reconstitution of zyxin with VASP binding site in zyxin-deficient hematopoietic progenitor cell-derived MKs restored GPIb-IX surface expression and proplatelet generation. Taken together, our findings identify zyxin as a regulator of platelet biogenesis and GPIb-IX surface expression through VASP-mediated cytoskeleton reorganization, suggesting possible pathogenesis of macrothrombocytopenia.

## Introduction

Platelets, the central regulator of thrombosis and hemostasis, are generated from the cytoplasm of megakaryocytes (MKs). In bone marrow (BM), hematopoietic stem cells differentiate into MKs in response to thrombopoietin (TPO) [1]. Immature MKs undergo endomitosis to increase their size and ploidy and develop a demarcation membrane system (DMS) that forms the plasma membrane of future platelets [2]. In the meantime, MK progenitors migrate from BM osteoblastic niche to the vascular niche [3, 4], in which proplatelets are formed and released from the matured MKs into the bloodstream. Finally, platelets are shed from proplatelets in the bloodstream.

The actin cytoskeleton plays an important role in platelet biogenesis [5]. Actin filaments localizing around the DMS network generate mechanical forces to initiate and mediate DMS formation [6]. Actin cytoskeleton reorganization guides the bending and bifurcating of the proplatelet shafts to increase the number of proplatelet ends [7]. Therefore, human mutations and gene-targeted mice deficient in the components of the actin cytoskeleton, such as non-muscle myosin heavy chain IIA (NMMHC-IIA), α-actinin, filamin A, tropomyosin 4, diaphanous-related formin 1 (DIAPH1), and tropomodulin 3, have shown thrombocytopenia due to abnormal platelet generation [8–13]. However, the molecular mechanisms underlying the complex process still remain elusive.

Zyxin is a focal adhesion protein wildly expressed in tissues [14]. Zyxin regulates actin cytoskeleton remodeling by providing docking sites for actin regulatory proteins [15–18]. The C-terminal of zyxin contains three LIM domains, which are essential for its localization to focal adhesions [18]. The N-terminus of zyxin contains the binding sites for actin filament crosslinker α-actinin [19], and displays the four proline-rich ActA repeats that can interact with the actin assembly modulator enabled (Ena)/vasodilator-stimulated phosphoprotein (VASP) proteins [20]. Mutations in α-actinin cause macrothrombocytopenia by inducing multiple defects of proplatelet formation [9]. We recently demonstrated that zyxin interacts with NMMHC-IIA [21], whose coding gene *MYH9* has the most macrothrombocytopenia-causing mutations [8]. Zyxin has been shown to express in platelets [14] and was found to be upregulated during megakaryocytic differentiation [22], whereas it is unclear whether zyxin plays a role in platelet biogenesis.

In the present study, we show that zyxin-deficient mice (*Zyx*^−/−^) display macrothrombocytopenia. We further demonstrate that zyxin, through mediating VASP subcellular localization, regulates actin cytoskeleton organization, GPIb-IX expression, and proplatelet generation.

## Results

### *Zyx*^−/−^ mice display macrothrombocytopenia

**Fig. 1:**
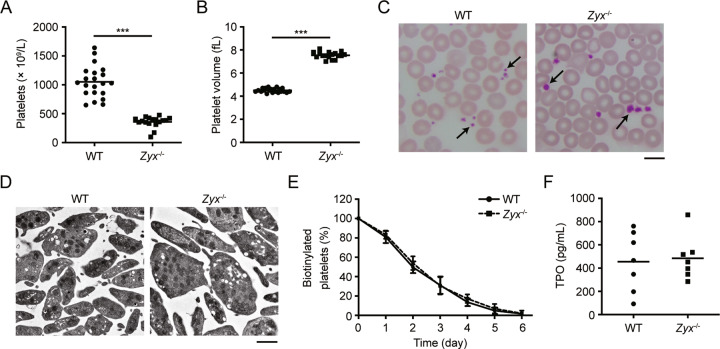
*Zyx*^−/−^ mice display macrothrombocytopenia. **A, B** Platelet count (**A**) and volume (**B**) were analyzed in whole blood from WT (*n* = 21) and *Zyx*^−/−^ (*n* = 17) mice. Means are indicated by horizontal lines. ***P < 0.001 compared with WT mice by Mann–Whitney test in (**A**) and unpaired Student’s *t*-test with Welch correction in (**B**). **C** Representative images of Wright–Giemsa stained blood smears from WT and *Zyx*^−/−^ mice (original magnification × 1,000). Scale bar: 10 μm. Platelets were indicated with arrows. **D** Representative transmission electron microscopy (TEM) images of WT and *Zyx*^−/−^ platelets (original magnification ×13,500). Scale bar: 1 μm. Images were obtained from five mice in each genotype in (**C**) and (**D**). **E** WT and *Zyx*^−/−^ mice were intravenously injected with NHS-biotin, and peripheral blood was taken at the indicated time points after injection. The percentage of biotinylated platelets was determined by flow cytometry; *n* = 10 mice per genotype. Data are expressed as means ± SD. **F** Serum TPO levels in WT and *Zyx*^−/−^ mice; *n* = 7 mice per genotype. Means are indicated by horizontal lines.

Zyxin has been demonstrated to express in both human and murine platelets [14, 23]. In order to investigate the role of zyxin in platelet biogenesis, we first performed a hematologic analysis in *Zyx*^−/−^ mice. Interestingly, we found that *Zyx*^−/−^ mice displayed severe macrothrombocytopenia, with a 65% reduction of platelet count and a 68% increase of platelet volume compared with wild-type (WT) mice (Fig. 1A, B). The *Zyx*^−/−^ platelets presented normal shape, but obviously enlarged size (Fig. 1C, D). In contrast, the number and morphology of leukocytes and red blood cells were not altered in *Zyx*^−/–^ mice (Table S1).

To investigate whether the thrombocytopenia is due to the enhanced platelet turnover, platelet lifespan was measured in *Zyx*^−/−^ mice. We found that platelet lifespan was not obviously altered in *Zyx*^−/−^ mice (*t*_1/2_ = 49 h for WT vs *t*_1/2_ = 51 h for *Zyx*^−/−^ mice) (Fig. 1E). In addition, the percentage of reticulated platelets was not altered, while the reticulated platelet number was markedly reduced in *Zyx*^−/−^ mice (Fig. S1). Since TPO is essential for MK proliferation and maturation, serum TPO level was examined. There were comparable levels of serum TPO between *Zyx*^−/−^ and WT mice (Fig. 1F). These data show that the macrothrombocytopenia in *Zyx*^−/−^ mice is not caused by accelerated platelet clearance or impaired TPO generation.

### Zyxin deficiency impairs MK maturation, DMS development, and proplatelet generation

**Fig. 2:**
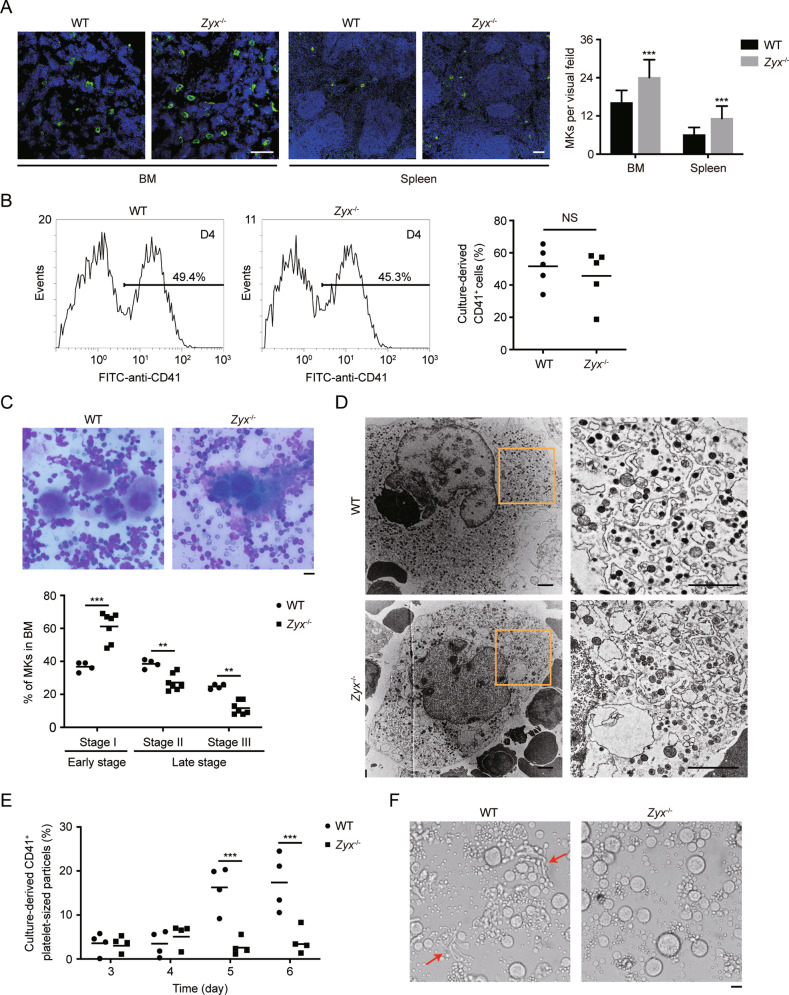
Normal MK differentiation but impaired maturation, DMS development, and proplatelet formation in *Zyx*^−/−^ MKs. **A** MK counts in mouse BM and spleen. Left panels, representative confocal images of MKs in femoral BM and spleen sections immunostained with anti-CD41 antibody. MKs (CD41) are in green, and nuclei are in blue. Original magnification × 200 for BM sections and × 100 for spleen sections. Scale bar: 100 μm. Right panel, the quantification of MK numbers per visual field; *n* = 15 visual fields from five mice per genotype. Data are expressed as means ± SD. **B** Percentage of CD41^+^ cells differentiated from WT and *Zyx*^−/−^ FL HPCs on day 4 was analyzed by flow cytometry. Data are from five independent experiments. **C** Upper panels, representative microscopy images of MKs in BM smears stained with Wright–Giemsa stain from WT and Zyx^−/−^ mice (original magnification × 400). Scale bar: 20 μm. Lower panel, the quantification of different stages of MKs using BM smears from WT (*n* = 4) and Zyx^−/−^ mice (*n* = 7). No less than 100 MKs were analyzed for each mouse. **D** Representative TEM images of MKs isolated from WT and *Zyx*^−/−^ mouse BM (original magnification, ×3,400 for left panels, and ×13,500 for right panels). Images were obtained from five mice in each genotype. Scale bar: 1 μm. **E** Percentage of CD41^+^ platelet-sized particles released from culture-derived MKs was analyzed by flow cytometry. Data are from four independent experiments. **F** Representative microscopy images of proplatelet formation from culture-derived MKs (**E**) on day 4 (original magnification ×200). Scale bar, 20 μm. Proplatelet protrusions were indicated with arrows. Means are indicated by horizontal lines in (**B**), (**C**), and (**E**). **P < 0.01, ***P < 0.001, compared with WT mice by unpaired Student’s *t*-test in (**A**) and (**B**), and by two-way ANOVA followed by Bonferroni’s post hoc test in (**C**) and (**E**). NS, not significant.

To investigate the mechanism for zyxin deficiency-mediated macrothrombocytopenia, we first characterized the megakaryopoiesis in *Zyx*^−/−^ mice. We found that, compared with WT mice, *Zyx*^−/−^ mice showed a 50% increase in MK number of the BM sections (16 MKs per visual field for WT vs 24 for *Zyx*^−/−^ mice, Fig. 2A). Spleen is also an important hematopoietic organ in mice. We found that there was an 83% increase in splenic MK number (6 MKs per visual field for WT vs 11 for *Zyx*^−/−^ mice, Fig. 2A). Therefore, the thrombocytopenia in *Zyx*^−/−^ mice does not result from a lack of MKs. We next examined the effect of zyxin deletion on MK differentiation in vitro by culturing mouse fetal liver hematopoietic progenitor cells (FL HPCs). The percentages of CD41 positive (CD41^+^) (Fig. 2B) and CD61 positive (CD61^+^) (Fig. S2) cells were comparable between WT and *Zyx*^−/−^ FL HPC cultures. We further cultured bone marrow hematopoietic progenitor cells (BM HPCs) from *Zyx*^−/−^ and WT mice. There was also not difference in the percentages of CD41^+^ and CD61^+^ cells between the two cultures (Fig. S3). These data suggest that zyxin is not required for MK differentiation.

We next investigated the effect of zyxin deficiency on MK maturation and proplatelet production. Giemsa-stained BM smears showed more Stage I MKs (immature MKs) and fewer Stage II and III MKs (mature MKs) in *Zyx*^−/−^ mice (Fig. 2C). The ultrastructure of Stage III MKs from BM aspirates was further analyzed by transmission electron microscopy (TEM). WT MKs showed well-organized DMS, but *Zyx*^−/−^ MKs showed disorganized DMS and a lot of vacuoles (Fig. 2D), suggesting the defective development of DMS in *Zyx*^−/−^ MKs.

Finally, platelet generation was examined in *Zyx*^−/−^ FL HPC-derived MKs. Culture-derived MKs started to release CD41-positive (CD41^+^) platelet-sized particles from day 5 (Fig. 2E). On day 5 and day 6, there were 13.6% and 11.8% CD41^+^ platelet-sized particles from WT MKs, while only 2.6% and 2.9% from *Zyx*^−/−^ MKs (Fig. 2E). Furthermore, Fig. 2F showed that proplatelet protrusions from *Zyx*^−/−^ MKs were obviously reduced compared with that from WT MKs. Taken together, these data show that zyxin deletion impairs MK maturation, DMS development, and proplatelet generation.

### Zyxin deficiency reduces glycoprotein (GP) Ib-IX levels in both platelets and MKs

**Fig. 3:**
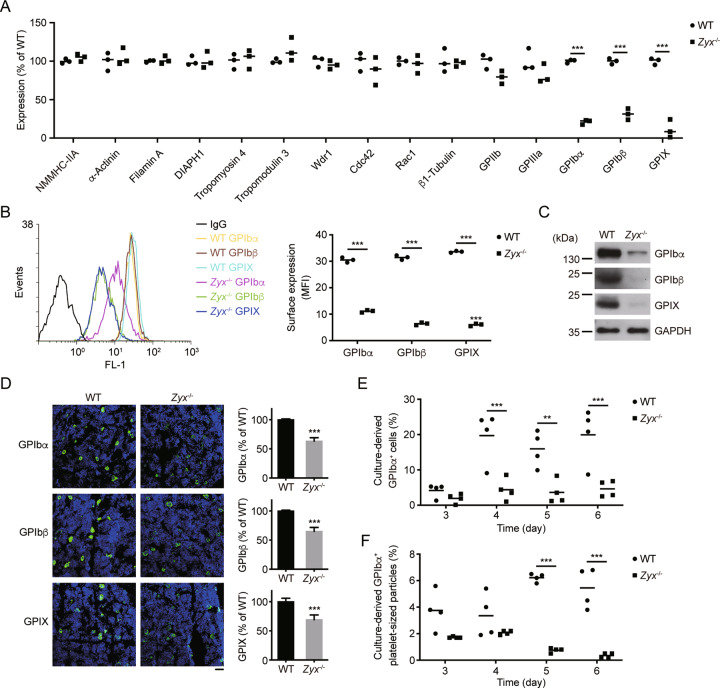
GPIb-IX expression is reduced in *Zyx*^-/-^ platelets and MKs. **A** Differential proteomic analysis of proteins expressed in WT and *Zyx*^−/−^ platelets; *n* = 3 mice per genotype. **B** Surface level of GPIb-IX complex on WT and *Zyx*^−/−^ platelets analyzed by flow cytometry; *n* = 3 mice per genotype. MFI, mean fluorescence intensity. **C** Western blot analysis of GPIb-IX in WT and *Zyx*^−/−^ platelets. Protein concentration has been adjusted to the same level between WT and *Zyx*^−/−^ platelet lysates. Blots are representative of five independent experiments. **D** Expression of GPIb-IX complex in WT and *Zyx*^−/−^ MKs. Left panels, representative confocal images of MKs in WT and *Zyx*^−/−^ mouse femoral BM sections immunostained with anti-GPIbα, GPIbβ, and GPIX antibodies (original magnification ×200). GPIbα, GPIbβ, and GPIX in MKs are in green, and nuclei are in blue. Scale bar: 100 μm. Right panels, the quantification of MFI of GPIbα, GPIbβ, and GPIX in the left panels analyzed by ImageJ software; *n* = 10 visual fields from 5 mice per genotype. Data are expressed as means ± SD. **E** Percentage of GPIbα^+^ cells differentiated from WT and *Zyx*^−/−^ FL HPCs was analyzed by flow cytometry. **F** Percentage of GPIbα^+^ platelet-sized particles released from culture-derived MKs in (**E**) was analyzed by flow cytometry. Data are from four independent experiments in (**E**) and (**F**). Means are indicated by horizontal lines in (**A**), (**B**), (**E**), and (**F**). **P < 0.01, ***P < 0.001, compared with WT mice by unpaired Student’s *t*-test in (**A**), (**B**), and (**D**), and by two-way ANOVA followed by Bonferroni’s post hoc test in (**E**) and (**F**).

As known, proteins related to the actin cytoskeleton or microtubular system have been reported to be associated with macrothrombocytopenia [8–13, 24–26]. In addition, deficiency or reduction of the major platelet membrane GPIb-IX and GPIIb/IIIa also cause macrothrombocytopenia named Bernard–Soulier syndrome (BSS) and *ITGA2B/ITGB3*-related thrombocytopenia, respectively [27, 28]. To further explore the molecular mechanism for zyxin deletion-induced macrothrombocytopenia, differential proteomic analysis was performed to compare the amount of the proteins associated with macrothrombocytopenia between WT and *Zyx*^−/−^ platelets. We found that GPIbα, GPIbβ, and GPIX, but not NMMHC-IIA, α-actinin, filamin A, DIAPH1, Tropomyosin 4, tropomodulin 3, Wdr1, Cdc42, Rac1, β1-tubulin, or GPIIb/IIIa, were significantly reduced in *Zyx*^−/−^ platelets (Fig. 3A).

We next checked the level of GPIb-IX in *Zyx*^−/−^ platelets. The surface levels of the three subunits were significantly decreased in *Zyx*^−/−^ platelets (a 63%, 80%, and 81% decrease in GPIbα, GPIbβ, and GPIX, respectively) (Fig. 3B); in contrast, GPIIb was increased by 40% (due to the enlarged size of the *Zyx*^-/-^ platelets) (Fig. S4). The total amounts of GPIbα, GPIbβ, and GPIX were also markedly reduced in *Zyx*^−/−^ platelets (Fig. 3C).

We further confirmed these findings by detecting platelet function. Consistently, botrocetin-induced platelet aggregation, which is mediated by the interaction of GPIbα and von Willebrand factor, was obviously reduced in *Zyx*^−/−^ platelets (Fig. S5A). Furthermore, *Zyx*^−/−^ platelets tend to disaggregate in response to 0.05 U/mL thrombin, consistent with the requirement of GPIbα in low dose thrombin-induced platelet activation [29]. However, zyxin ablation did not affect platelet aggregation induced by relatively high doses of thrombin (0.1 U/mL and 0.2 U/mL) (Fig. S5B). In addition, there was no significant difference in ADP- or U46619-induced platelet aggregation between WT and *Zyx*^−/−^ platelets (Fig. S5C, D). These data suggest that zyxin deficiency impairs only GPIb-IX-dependent platelet function.

We also examined the expression levels of GPIb-IX/GPIbα in MKs. The mean fluorescence intensities (MFI) of GPIbα, GPIbβ, and GPIX were significantly decreased in *Zyx*^−/−^ MKs in the immunostained BM sections (Fig. 3D). The GPIbα−positive (GPIbα^+^) cells were markedly reduced in *Zyx*^−/−^ FL HPC-derived MKs (Fig. 3E). Moreover, the GPIbα^+^ platelet-sized particles were also reduced (Fig. 3F). These data indicate that zyxin deficiency results in the reduction of GPIb-IX in both platelets and MKs.

### Zyxin knockdown results in lysosome-mediated GPIbα degradation in human megakaryocytic Dami cells

**Fig. 4:**
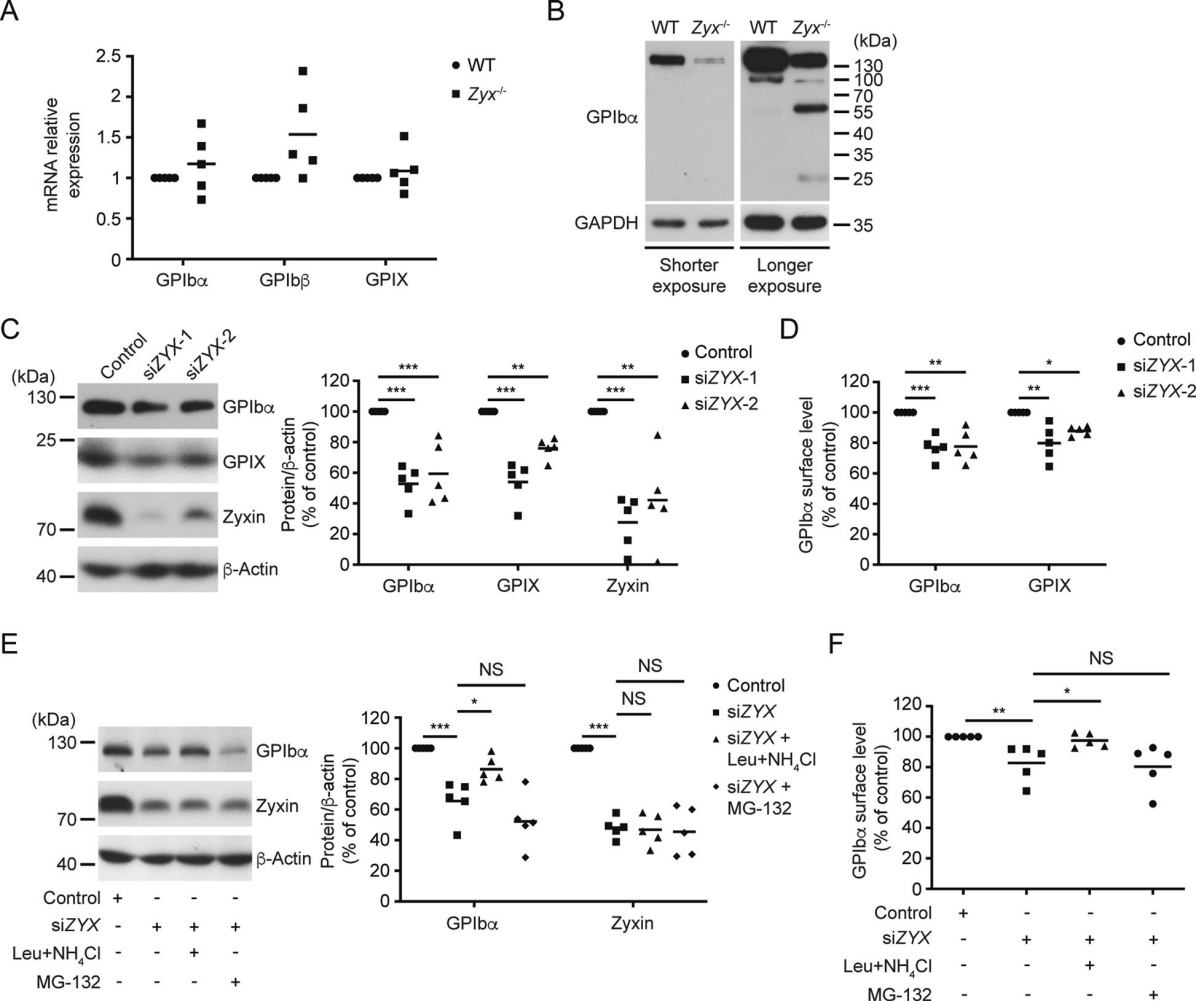
Knockdown of zyxin results in lysosome-mediated GPIbα degradation. **A** mRNA levels of GPIbα, GPIbβ, and GPIX in WT and *Zyx*^−/−^ platelets. mRNA expression was analyzed by qRT-PCR and determined by a ratio relative to the control GAPDH. The data were expressed as the ratio relative to WT; *n* = 5 mice per genotype. **B** Western blot analysis of GPIbα protein in WT and *Zyx*^−/−^ platelets. Protein concentration was adjusted to the same level between WT and *Zyx*^−/−^ platelet lysates. Blots are representative of five independent experiments. **C, D** Dami cells were transfected with siRNAs targeting zyxin (si*ZYX*-1, and -2) or negative control siRNA (control). The expression of GPIbα and GPIX in Dami cells was analyzed by Western blot; the blots are representative of five independent experiments (**C**). The surface level of GPIbα and GPIX was analyzed by flow cytometry (**D**). **E, F** Dami cells were treated with or without 10 μg/mL leupeptin plus 10 mM NH_4_Cl (Leu + NH_4_Cl) and MG-132 (100 nM) for 12 h after zyxin siRNA (si*ZYX*-1) transfection. The total expression of GPIbα was analyzed with Western blot; the blots are representative of five independent experiments (**E**). The surface level of GPIbα was analyzed by flow cytometry (**F**). Data are from five independent experiments in (**C**–**F**). Means are indicated by horizontal lines in (**A**) and (**C–F**). *P < 0.05, **P < 0.01, ***P < 0.001, by one-way ANOVA followed by Dunnett’s post hoc test in (**C–F**). NS, not significant.

GPIb-IX complex is assembled in the endoplasmic reticulum, transported into Golgi, and expressed on the plasma membrane [30]. The presence of all three subunits and proper transportation are required for the efficient surface expression of the whole complex [31]. To explore the reason for the defective GPIb-IX expression in *Zyx*^−/−^ platelets, we first examined whether zyxin deletion reduced transcription of these three genes. The quantitative real-time PCR (qRT-PCR) results showed that the mRNA levels of the three subunits did not decrease in *Zyx*^−/−^ platelets (Fig. 4A), excluding the possibility of defective gene transcription. Further analysis of GPIbα by Western blot showed additional anti-GPIbα antibody-positive bands (60 kDa and 25 kDa) in *Zyx*^−/−^ platelets (Fig. 4B), suggesting that GPIb-IX complex may be degraded in *Zyx*^−/−^ platelets.

We next used a human megakaryocytic cell line, Dami cells, which express the GPIb-IX complex on the cell surface [32], to explore the role of zyxin in GPIb-IX expression. The small interfering RNAs (siRNAs) targeting zyxin were transfected into Dami cells to knockdown zyxin. We first verified that the zyxin siRNAs significantly reduced the mRNA level of zyxin but not that of GPIbα, GPIbβ, or GPIX (Fig. S6). Zyxin siRNAs reduced zyxin expression, and in the meantime, the levels of GPIbα and GPIX were obviously reduced (Fig. 4C). Moreover, GPIbα and GPIX surface levels were significantly decreased (Fig. 4D). These data, consistent with the data from *Zyx*^−/−^ platelets and MKs, reveal that knockdown of zyxin reduces GPIb-IX level in human megakaryocytic Dami cells.

GPIbα was reported to be degraded through a lysosome-dependent manner [30]. We treated Dami cells with lysosomal inhibitors (leupeptin + NH_4_Cl) and a proteasome inhibitor (MG-132) after zyxin siRNA transfection. As shown in Fig. 4E and F, inhibition of lysosomes but not proteasomes restored GPIbα surface expression as well as the total level of full-length GPIbα. We further used another lysosomal inhibitor (bafilomycin A1) to verify this. Bafilomycin A1 indeed rescued zyxin knockdown-induced GPIbα reduction (Fig. S7). These data demonstrate that zyxin deficiency results in lysosome-mediated GPIbα degradation in the human megakaryocytic cell line.

### Zyxin deficiency enhances microtubular ring in platelets

**Fig. 5:**
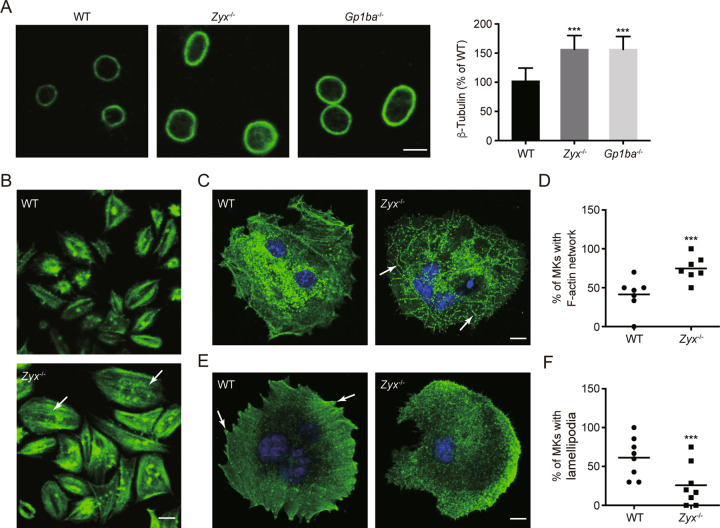
Zyxin deficiency affects microtubule and actin cytoskeleton organization. **A** Left panels, representative confocal images of resting WT, *Zyx*^−/−^ and *Gp1ba*^−/−^ platelets stained for β-tubulin (green). Scale bar: 3 μm. Right panel, the quantification of MFI of β-tubulin on the left was analyzed by ImageJ software; *n* = 10 visual fields from five mice per genotype. Data are expressed as means ± SD. **B** Confocal images of WT and *Zyx*^−/−^ platelets were allowed to spread on fibrinogen in the presence of thrombin and stained for F-actin (green). Scale bar: 3 μm. Images are representative of five independent experiments. **C, D** MKs cultured from mouse FL HPCs were allowed to spread on type I collagen. Representative confocal images of spread WT and *Zyx*^−/−^ MKs stained for F-actin (green) and nuclei (blue); scale bar: 10 μm (**C**). The percentage of MKs with the organized F-actin network along collagen fibers (as indicated by arrows in **C**) (**D**). No less than 60 MKs per genotype from seven independent experiments were analyzed. **E, F** MKs cultured from mouse FL HPCs were allowed to spread on fibrinogen. Representative confocal images of spread WT and *Zyx*^−/−^ MKs stained for F-actin (green) and nuclei (blue); scale bar: 10 μm (**E**). The percentage of MKs with lamellipodia (as indicated by arrows in **E**) (**F**). No less than 60 MKs per genotype from eight independent experiments were analyzed (**C–F**). The original magnification of all the images is ×630. Means are indicated by horizontal lines in (**D**) and (**F**). ***P < 0.001, compared with WT mice by one-way ANOVA followed by Dunnett’s post hoc test in (**A**) and by unpaired Student’s *t*-test in (**D**) and (**F**).

Microtubules play critical roles in the extension of proplatelets [5, 7]. The proplatelets from a mouse model of BSS contained twice as many tubulin fibers in the marginal microtubular ring [33]. Thus, we also examined the microtubular system in *Zyx*^−/−^ platelets. First, we constructed a mouse model of BSS by deletion of GPIbα (Fig. S8). The GPIbα-deficient (*Gp1ba*^-/-^) mice presented the typical macrothrombocytopenia phenotype (Table S2). The same as the finding in *Gp1ba*^−/−^ platelets, a brighter and thicker marginal band was found in *Zyx*^−/−^ platelets compared with that in WT platelets (Fig. 5A). These data suggest that the phenomenon of *Zyx*^−/−^ platelets is similar to that of the platelets from a mouse model of BSS [32].

### Zyxin deficiency alters actin cytoskeleton organization

It has been implied that actin cytoskeleton organization is essential for appropriate insertion or removal of membrane proteins [34]. And alteration of actin cytoskeleton reorganization impairs proplatelet formation leading to macrothrombocytopenia [5]. Zyxin regulates actin assembly [16–18]. Therefore, zyxin ablation may alter actin cytoskeleton organization, contributing to defective GPIb-IX complex expression and proplatelet generation. To test this hypothesis, F-actin organization was visualized by phalloidin staining in platelets spreading on the fibrinogen surface. The stress fibers were bundled along the sides of the WT platelets; in contrast, the stress fibers were disseminated in the whole cytoplasm of *Zyx*^−/−^ platelets (Fig. 5B). We further detected actin cytoskeleton in MKs from WT and *Zyx*^−/−^ mice. In MKs spreading on immobilized collagen, only 41% of WT MKs displayed an organized F-actin network along collagen fibers, whereas 75% of *Zyx*^−/−^ MKs presented the phenomena (Fig. 5C, D). Furthermore, for MKs spreading on immobilized fibrinogen, there was 61% of WT MKs displaying the lamellipodia, whereas this was only seen in about 26% of *Zyx*^−/−^ MKs (Fig. 5E, F). The spread areas were not affected by zyxin deficiency on both collagen and fibrinogen surfaces (Fig. S9). These data indicate that actin cytoskeleton organization is altered in *Zyx*^−/−^ platelets and MKs.

### Zyxin deficiency results in VASP mislocalization

**Fig. 6:**
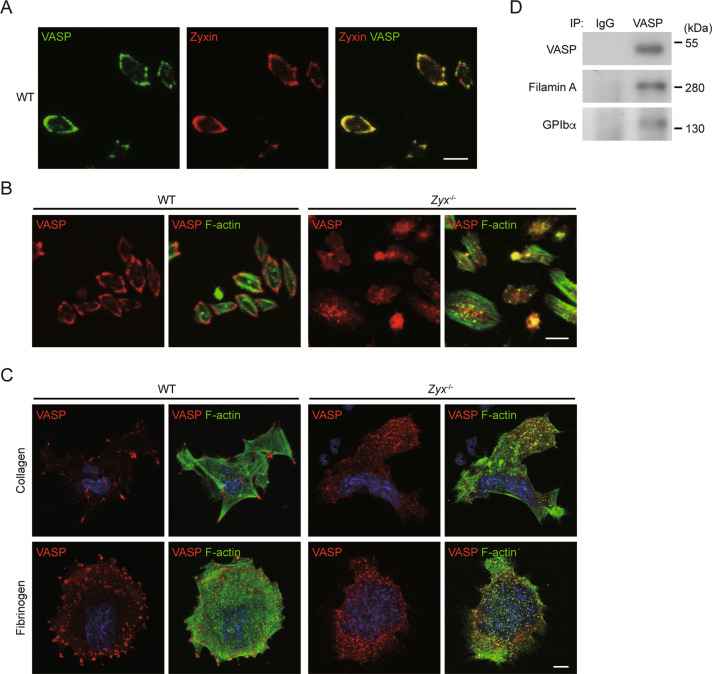
Zyxin deficiency affects VASP localization. **A, B** Platelets were allowed to spread on fibrinogen in the presence of thrombin. Representative confocal images of spread WT platelets stained for VASP (green) and zyxin (red) (**A**). Representative confocal images of spread WT and *Zyx*^−/−^ platelets stained for and VASP (red) and F-actin (green) (**B**). Scale bar: 5 μm. **C** Representative confocal images of WT and *Zyx*^−/−^ FL HPC-derived MKs allowed to spread on type I collagen and fibrinogen and stained for VASP (red), F-actin (green), and nuclei (blue). Scale bar: 10 μm. The original magnification of all the images is ×630. Results are representative of five independent experiments. **D** Filamin A and GPIbα were co-immunoprecipitated with VASP by anti-VASP antibody in WT platelet lysate. Results are representative of five independent experiments.

We next explored the mechanism for zyxin in regulating actin cytoskeleton organization. α-Actinin and VASP are two important zyxin binding partners [19, 20]. We recently identified NMMHC-IIA as a zyxin binding protein [21]. The three proteins all exist in platelets and play important roles in actin cytoskeleton reorganization. Zyxin deletion did not affect the expression of the three proteins (Fig. 3A, Fig. S10A). In the absence of zyxin, the distributions of α-actinin and NMMHC-IIA were not obviously altered in platelets spreading on the fibrinogen surface (Fig. S10B). Moreover, zyxin deficiency did not affect the phosphorylation of NMMHC-IIA at Ser1916 and Ser1943, which is related to the activity of NMMIIA [35], in the platelets either under basal condition or stimulated with various agonists (Fig. S11). In contrast, we found that zyxin colocalized with VASP (Fig. 6A) in WT platelets, and the distribution of VASP was obviously disrupted in *Zyx*^−/−^ platelets compared with that in WT platelets (Fig. 6B), suggesting the essential role for zyxin in VASP localization. Next, we verified this finding in MKs. Consistent with the finding in platelets, VASP was localized at focal adhesions (Fig. 6C), which is the same as zyxin in WT MKs (Fig. S12). Without zyxin, VASP was distributed all over the cells in *Zyx*^−/−^ MKs (Fig. 6C). These data suggest that zyxin is essential for subcellular VASP localization in platelets and MKs.

Next, we explored the mechanism for zyxin in regulating GPIb-IX surface expression. As known, VASP is an actin regulatory protein. Filamin A that crosslinks actin filaments associates with the cytoplasmic domain of GPIbα anchoring the GPIb-IX complex to the membrane skeleton for surface expression. Therefore, we hypothesized that there might be an association between VASP and filamin A or GPIbα. Indeed, we find that VASP could be co-immunoprecipitated with both filamin A and GPIbα (Fig. 6D). These findings suggest the possibility that zyxin, through VASP, regulates GPIb-IX surface expression.

### The interaction of zyxin with VASP is required for GPIbα surface expression and platelet production

**Fig. 7:**
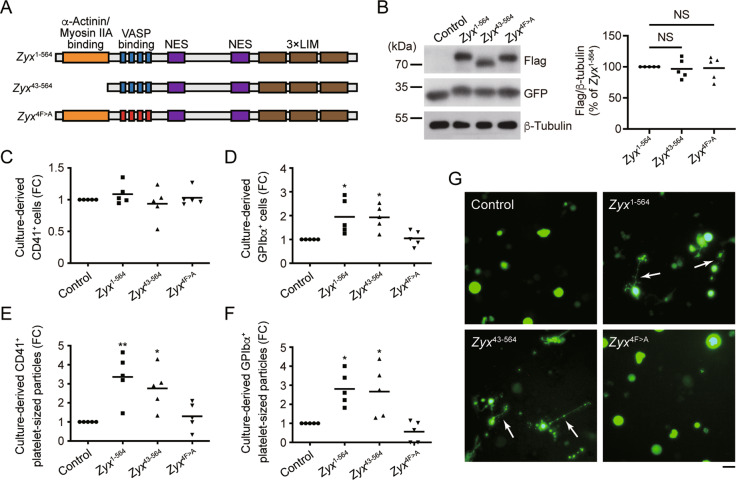
The interaction of zyxin with VASP is required for zyxin-mediated GPIbα expression and platelet production. **A** Cartoons for the constructs of zyxin mutants lacking α-actinin and NMMHC-IIA (*Zyx*^43–564^), and VASP (*Zyx*^4F > A^) binding sites. NES, nuclear export signals. **B** *Zyx*^−/−^ FL HPCs were infected with adenoviruses expressing WT zyxin (*Zyx*^1–564^), zyxin mutants *Zyx*^43-564^ and *Zyx*^4F > A^, and vehicle vector (control). The expression levels of WT zyxin and zyxin mutants were determined by Western blot with an anti-flag tag antibody. The green fluorescence (GFP) indicates the introduction of the plasmids into HPC-derived MKs. Blots are representative of five independent experiments. **C–F** The expression of CD41 (**C**) and GPIbα (**D**) on culture-derived MKs was determined on day 4 by flow cytometry. The presence of CD41^+^ (**E**) and GPIbα^+^ (**F**) platelet-sized particles released from culture-derived MKs was determined on day 5 by flow cytometry. FC, fold-change. Data are from five independent experiments, and means are indicated by horizontal lines in (**B**–**F**). *P < 0.05, **P < 0.01, compared with control by one-way ANOVA followed by Dunnett’s post hoc test. NS, not significant. **G** Representative fluorescent microscopy images of proplatelet formation from five independent experiments (original magnification ×200). Scale bar: 40 μm. Proplatelet protrusions were indicated with arrows.

We further investigated whether zyxin regulates GPIb-IX expression and platelet production through the interaction with VASP. The binding sites for α-actinin and NMMHC-IIA are located in the N-terminal 42 amino acids of zyxin [19, 21], and the binding site for VASP is in the four proline-rich ActA repeats in zyxin (Fig. 7A) [20]. In order to distinguish which site is essential for GPIb-IX expression and platelet production, we constructed zyxin mutants lacking binding sites for α-actinin and NMMHC-IIA (*Zyx*^43–564^), and VASP (*Zyx* ^4F > A^) (Fig. 7A). *Zyx*^–/−^ FL HPCs were infected with adenoviruses expressing WT zyxin and zyxin mutants, and similar expression levels of these genes were verified with Western blot (Fig. 7B). The expressions of WT zyxin (*Zyx*^1–564^) and zyxin mutants (*Zyx*^43–564^ and *Zyx* ^4F > A^) in *Zyx*^−/−^ FL HPCs did not affect the percentage of CD41^+^ cells compared with that of vector control (Fig. 7C), suggesting that these genes did not affect the MK differentiation. However, WT zyxin and *Zyx*^43–564^ significantly increased the percentage of GPIbα^+^ cells; in contrast, *Zyx*^4F > A^ did not have the effect (Fig. 7D), indicating that WT and *Zyx*^43–564^ but not *Zyx*^4F > A^ could rescue GPIbα surface expression in *Zyx*^−/−^ FL HPC-derived MKs. Consistently, WT zyxin and *Zyx*^43–564^ but not *Zyx*^4F > A^ enhanced CD41^+^ platelet-sized particles and GPIbα^+^ platelet-sized particles from *Zyx*^−/−^ FL HPC-derived MKs (Fig. 7E, F). Moreover, Fig. 7G showed that the expression of WT zyxin and *Zyx*^43–564^ but not *Zyx*^4F > A^ promoted proplatelet formation. These data suggest that the interaction of zyxin with VASP is essential for GPIbα surface expression and platelet production.

## Discussion

In this study, we report for the first time that zyxin is essential for platelet biogenesis and GPIb-IX surface expression. Our data show that: (1) *Zyx*^−/−^ mice display macrothrombocytopenia, (2) zyxin is essential for DMS formation and proplatelet production, (3) zyxin ablation results in GPIbα degradation leading to reduction of GPIb-IX surface expression and GPIb-IX-dependent platelet function, (4) deletion of zyxin alters actin cytoskeleton organization, and (5) the interaction of zyxin with VASP is required for proplatelet production and GPIb-IX surface expression.

Defective proplatelet formation has been reported in more than ten forms of inherited thrombocytopenia [36]. Most of these diseases are macrothrombocytopenia caused by mutations in genes encoding for components of the actomyosin cytoskeleton or microtubular system [36]. Reorganization of the actin cytoskeleton is crucial for DMS formation or extension and release of proplatelets [6, 7]. Therefore, defect in genes associated with the actomyosin cytoskeleton, such as *MYH9* and *ACTN1*, results in alteration of proplatelet formation [8–11]. Microtubules composed of α- and β-tubulin are the driving force of proplatelet elongation [5, 7]. Therefore, defects in *TUBB1* induce abnormalities of microtubule function, affecting proplatelet formation [26]. In line with the reported macrothrombocytopenia, we found that the absence of zyxin resulted in actin cytoskeleton disorganization and enhanced tubulin filaments in the marginal band. Thus, our data support that zyxin deficiency alters the actin cytoskeleton and microtubular system resulting in defective proplatelet formation and macrothrombocytopenia.

Zyxin is essential for actin filament remodeling. Our data showed that zyxin deficiency affects F-actin organization. Without zyxin, VASP is disseminated in the whole platelets. VASP is capable of bundling actin filaments and inducing actin polymerization [37, 38]. The dislocated VASP may induce actin polymerization in altered places, leading to altered organization of actin skeleton in *Zyx*^−/−^ platelets and MKs. Therefore, it is reasonable to find that the actin filaments were disorganized in *Zyx*^−/−^ platelets and MKs, resulting in defective proplatelet formation. Consistently, reconstitution of zyxin lacking VASP binding site in *Zyx*^−/−^ FL HPCs did not restore platelet production.

Interestingly, we found that zyxin deficiency diminished GPIb-IX surface expression both in *Zyx*^−/−^ platelets and MKs. These findings suggest that the molecular mechanism of zyxin deficiency-induced macrothrombocytopenia may be similar to that of BSS. GPIb-IX complex is assembled in the endoplasmic reticulum and traffics to the plasma membrane for surface expression [30]. Filamin A that binds to actin associates with the cytoplasmic domain of GPIbα anchoring the GPIb-IX complex for surface expression. One of the roles of actin is for membrane trafficking [39]. Therefore, actin organization might be critical for GPIb-IX trafficking and surface expression. In the current study, the data showed that VSAP localized with zyxin, and the interaction of zyxin with VASP is required for GPIb-IX surface expression. Thus, it is most likely that zyxin through VASP-mediated actin reorganization regulates GPIb-IX trafficking and surface expression. According to this theory, loss of zyxin results in actin cytoskeleton disorganization, which impairs GPIb-IX trafficking to the membrane and incurs GPIbα degradation. In support of this, GPIbα degradation bands were observed in *Zyx*^−/−^ platelets, and knockdown of zyxin in Dami cells results in lysosome-mediated degradation of GPIbα. GPIb-IX complex, the receptor for the von Willebrand factor, plays an important role in thrombosis and hemostasis. This is the first time we have disclosed that zyxin regulates platelet function through regulating the GPIb-IX surface expression.

We selected a human megakaryocytic cell line, Dami cells, to demonstrate our findings. Consistent with the findings with *Zyx*^−/−^ mouse platelets and MKs, knockdown of zyxin also reduced GPIb-IX surface level in Dami cells. These data provide important evidence that deletion of zyxin in human cells causes the same effects as it does in mice.

We found that *Zyx*^−/−^ platelets showed brighter and thicker marginal microtubular rings. This is consistent with the findings that microtubules were obviously increased in GPIbα and filamin A null platelets, which also showed defective GPIb-IX surface expression [33, 40]. Furthermore, increased microtubules were observed in giant platelets from patients with May–Hegglin anomaly [41]. These findings suggest the possible relevance of GPIbα to the microtubular system. Future study is needed to disclose the relationship between them.

Therefore, these findings delineate the mechanism of zyxin in platelet biogenesis and GPIb-IX expression. In the absence of zyxin, (1) VASP was mislocalized, leading to disorganization of actin cytoskeleton, (2) GPIb-IX could not be properly transported to the plasma membrane for surface expression, (3) microtubule filaments were elevated in the marginal ring. These factors independently or mutually promoting impair proplatelet formation which results in macrothrombocytopenia.

The relevance of zyxin with diseases has been reported [42, 43]. Here we show that the absence of zyxin incurs macrothrombocytopenia. Macrothrombocytopenia could cause severe or life-threatening hemorrhage. However, due to the pathogenic mechanism is not totally understood, many patients with macrothrombocytopenia still have not been diagnosed or were misdiagnosed [36]. In this sense, the current study not only identifies the role of zyxin in the pathogenesis of macrothrombocytopenia, more importantly, but also suggests that defect of zyxin or other cytoskeleton proteins may have the potential to incur macrothrombocytopenia in humans as well. Thus, the finding expands our horizons in finding novel macrothrombocytopenia-causing genes.

In conclusion, our study demonstrates that zyxin ablation results in macrothrombocytopenia in mice. Zyxin, through VASP-mediated cytoskeleton reorganization, regulates proplatelet formation and GPIb-IX surface expression. Our findings help to understand the mechanism of platelet biogenesis and suggest possible pathogenesis of macrothrombocytopenia.

## Materials and methods

### Mice

C57BL/6 WT mice were purchased from JOINN Laboratories (Beijing, China). *Zyx*^−/−^ mice were purchased from The Jackson Laboratory (Bar Harbor, ME) [44] and interbred with WT C57BL/6 mice to generate *Zyx*^−/−^ and WT control littermate mice.

The *Gp1ba*^−/−^ mouse model was established by CRISPR/Cas9 genome editing technology on a C57BL/6 J background to induce two double-stranded breaks flanking exon 2 of *Gp1ba*. The optimized sgRNAs were constructed in the pT7-sgRNA plasmid backbone, and then were in vitro transcribed. The sequences of two independent guide RNAs targeting exon 2 of the *Gp1ba* gene were gRNA1 5'-TCTCACAGTTTACTTCCAGC −3' and gRNA2 5'- TATTGGGCACAGTGGGCATT −3'. The in vitro-transcribed Cas9 mRNA together with two sgRNAs were microinjected into the cytoplasm of C57BL/6 J zygote. In total, 145 healthy zygotes were transferred to pseudopregnant recipients and allowed to develop to term. Genotyping was performed by PCR amplification across the targeted region (F: 5′-AGAAGCTCTGTTCCTCCAAAGGAC-3′, R: 5′-GGTAGTAGTGACCATGTAGCCTGAC −3′) to screen the born 15 pups. In total, ten founder mice were initially established and confirmed with the right PCR product (597 bp). The founder mice were further bred to WT C57BL/6 J mice to generate heterozygous *Gp1ba*-knockout mice and then were intercrossed to generate homozygous *Gp1ba*^−/−^ mice.

Mice were 6–12 weeks old, and experiments included balanced groups of male and female mice. All animal experiments complied with the regulatory standards of and were approved by the Ethics Committee of the First Affiliated Hospital of Soochow University.

### Antibodies and reagents

FITC-conjugated rat anti-mouse CD41 antibody (MWReg30, 553848), mouse BD Fc Block (anti-mouse CD16/32, clone 2.4G2, 553142), and propidium iodide (PI, 556463) were purchased from BD Biosciences (San Jose, CA, USA). FITC-conjugated streptavidin (405202), APC-conjugated (133914), and PE-conjugated (133906) rat anti-mouse CD41 antibodies (MWReg30) were purchased from Biolegend (San Diego, CA, USA). Rat anti-mouse GPIbα (Xia.G5, M040-0; Xia.G7, M042-0) and GPIbβ (Xia.C3, M050-0) antibodies, FITC-conjugated rat anti-mouse GPIbα (Xia.G5, M040-1), GPIbβ (Xia.C3, M050-1), and GPIX (Xia.B4, M051-1) antibodies, and DyLight 649-conjugated rat anti-mouse GPIbα antibody (Xia.G5, M040-3) were purchased from Emfret Analytics (Eibelstadt, Germany). Antibodies against β-actin (4970), GAPDH (5174), β-tubulin (2146), α-actinin (3134), VASP (3132), non-muscle myosin heavy chain IIA (NMMHC-IIA, 3403), phosphor-NMMHC-IIA (Ser1943) (5026), and filamin A (4762) were from Cell Signaling Technology (Beverly, MA, USA). Antibodies against zyxin (10330-1-AP), GPIX (14-564-1-AP), GFP tag (50430-2-AP), and flag tag (20543-1-AP) were from Proteintech (Wuhan, China). Rabbit anti-phosphor-NMMHC-IIA (Ser1916) was purchased from ECM Biosciences (Versailles, KY, USA). The antibody for Western blot against mouse GPIX (GTX32502) was from GeneTex (Irvine, CA, USA). HRP-conjugated goat anti-rabbit IgG (A0208), anti-mouse IgG (A0216), and anti-rat IgG (A0192) secondary antibodies, and 2-(4-Amidinophenyl)-6-indolecarbamidine dihydrochloride (DAPI) were from Beyotime Institute of Biotechnology (Shanghai, China). Rabbit anti-mouse GPIbα antibody (PAB108Mu01) for Western blot was from Cloud-Clone Corp (Houston, TX, USA). Thrombopoietin (TPO) mouse ELISA kit (ab100748), rat anti-mouse CD41 antibody (MWReg30, ab33661), and FITC-conjugated goat anti-mouse antibody (ab6785) were purchased from Abcam (Cambridge, MA, USA). Mouse anti-zyxin antibody (sc-293448) and Protein G Plus Agarose (sc-2002) were from Santa Cruz Biotechnology (Santa Cruz, CA, USA). BCA Protein Assay Kit (23225), Lipofectamine™ 2000 Transfection Reagent (11668-027), Alexa Fluor 488-conjugated phalloidin (A12379), flow cytometry staining buffer (T00-4222-26), Alexa Fluor 555-conjugated goat anti-rat IgG (A21434), Alexa Fluor 555-conjugated goat anti-rabbit IgG (A21428), Alexa Fluor 488-conjugated goat anti-rabbit IgG (A11008), and Alexa Fluor 488-conjugated goat anti-mouse IgG (A11001) secondary antibodies were purchased from Thermo Fisher Scientific (Rockford, IL, USA). N-hydroxysuccinimide biotin (NHS-biotin) (H1759), ADP (A2754), and human thrombin (T6884) were purchased from Sigma (St. Louis, MO, USA). U46619 (538944) and human fibrinogen (341576) were purchased from Calbiochem (La Jolla, CA, USA). Collagen (385) was from Chrono-log Corp (Havertown, PA, USA). Anti-human GPIX antibody (clone FMC25) (MAB1202) was from Millipore (Darmstadt, Germany). EasySep buffer (20144), mouse hematopoietic progenitor cell isolation kit (19856), and Stemspan^TM^ Serum-Free Expansion Medium (SFEM) (09650) were purchased from STEMCELL Technologies (Vancouver, Canada). Mouse TPO (315-14) was from PeproTech (Rocky Hill, NJ, USA). Bafilomycin A1 (HY-100558) was from MedChemExpress (Princeton, NJ, USA). Leupeptin hemisulfate (M3636) and MG-132 (M1902) were from Abmole (Houston, TX, USA). Mouse anti-human GPIbα monoclonal antibody 6F3' was generated in our laboratory. Botrocetin was a kind gift from Dr. Renhao Li (Emory University).

### Hematologic analysis

Murine whole blood was collected from the postorbital veins and anti-coagulated with 1/7 volume of acid-citrate-dextrose (ACD, 2.5% trisodium citrate, 2.0% D-glucose, 1.5% citric acid). Platelet and blood cell counts were performed with Mindray BC-5000vet Hematologic Analyzer (Mindray Corporation, Shenzhen, China).

### Platelet preparation

For the preparation of washed mouse platelets, whole blood from mice was collected from postorbital veins or inferior vena cava using 1/7 volume of ACD as the anticoagulant. Platelets were washed with CGS buffer (0.123 M NaCl, 0.033 M D-glucose, 0.013 M trisodium citrate, pH 6.5) and resuspended in Modified Tyrode’s buffer (2.5 mM Hepes (N-2-hydroxyethylpiperazine-N’-2-ethanesulfonic acid), 150 mM NaCl, 2.5 mM KCl, 12 mM NaHCO_3_, 5.5 mM D-glucose, 1 mM CaCl_2_, 1 mM MgCl_2_, pH 7.4) to a concentration of 3 × 10^8^/mL and allowed to incubate at 22 °C for 1–2 h. For preparation of mouse platelet-rich plasma (PRP), whole blood was anti-coagulated with 1/9 volume of 3.8% trisodium citrate, and PRP were obtained by 100 × g centrifugation.

### Platelet and MK imaging

For blood smear, the murine whole blood from postorbital veins anti-coagulated with 1/7 volume of ACD was made into monolayer cells on a glass slide. For BM smear, the femoral BM was harvested and smeared on a glass slide with fetal bovine serum added in advance. Blood and BM smears were then stained with Wright–Giemsa Stain solution and examined by optical microscopy.

For TEM analysis of platelets, washed mouse platelets were centrifuged at 600 × g for 2 min and then fixed in 2.5% glutaraldehyde at 4 °C overnight. For TEM analysis of MKs, femora and tibias of mice were isolated and the BM was flushed out by 0.01 M phosphate-buffered saline (PBS) and then centrifuged at 300 × g for 2 min. The platelets or BM pellets were washed with PBS, postfixed in 1.0% osmium tetroxide for 1 h, gradually dehydrated using acetone, and then stained with saturated uranyl acetate. The samples were infiltrated, embedded with resin, and polymerized. Finally, ultrathin sections were observed with a transmission electron microscope (Philips CM 120, Eindhoven, The Netherlands).

### Platelet aggregation

Platelets in PRP or washed platelets (3 × 10^8^/mL) were stimulated with different concentrations of different agonists. Platelet aggregation was recorded in a Chrono-Log lumi-aggregometer at a stirring speed of 1200 rpm at 37 °C. Platelet aggregation was monitored continuously over 10 min.

### Platelet lifespan analysis

WT and *Zyx*^−/−^ mice were intravenously injected with 600 μg NHS-biotin in buffer containing 140 mM NaCl and 10% DMSO. Whole blood was collected by capillary tube from the retro-orbital venous plexus at various time points and mixed with ACD. Whole blood was stained by PE-conjugated anti-mouse CD41 antibody and FITC-conjugated streptavidin at room temperature (RT) for 1 h. The percentage of biotinylated platelets was determined by FC 500 flow cytometer (Beckman Coulter, Miami, FL, USA).

### Serum TPO measurement

Mouse blood was collected from postorbital veins and then incubated at 37 °C for 1 h and centrifuged to obtain the serum. Mouse serum TPO levels were measured according to the manufacturer’s protocol. Briefly, 50 μL serum was diluted with 50 μL assay buffer and added to the 96-well plate which has been coated with anti-mouse TPO antibody. After incubation, TPO was detected with biotinylated anti-TPO antibody and HRP-conjugated streptavidin. The plate was read at 450 nm in a Variskan Flash spectral scanning multimode reader (Thermo Fisher Scientific).

### Immunofluorescence staining on femora and spleen cryosections

Mouse femora were isolated, fixed with 4% paraformaldehyde (PFA), decalcified with decalcifying solution (2% PFA, 5% formic acid), and then dehydrated in 20% sucrose. Subsequently, the femora were embedded in Cryo-Gel and frozen at −80 °C. Eight-micrometer-thick cryosections were generated, fixed in 4% PFA, and blocked in 5% bovine serum albumin (BSA)/PBS at RT for 1 h. For MK counting in mouse femoral cryosections, MKs were labeled with anti-CD41 antibody (MWReg30, 10 μg/mL) at RT for 2 h and Alexa Fluor 555-conjugated goat anti-rat antibody (5 μg/mL) at RT for 1 h. For GPIb-IX complex expression detection in femoral MKs, anti-GPIbα (Xia.G5, 10 μg/mL), anti-GPIbβ (Xia.C5, 10 μg/mL) and anti-GPIX (10 μg/mL, GeneTex) antibodies were incubated overnight at 4 °C and Alexa Fluor 555-conjugated goat anti-rat (5 μg/mL) and Alexa Fluor 555-conjugated goat anti-rabbit (5 μg/mL) secondary antibodies were incubated at RT for 1 h. Cell nuclei were stained with 5 μg/mL DAPI. Samples were visualized with a Leica TCS SP8 confocal microscope (Leica Microsystems, Wetzlar, Germany).

For MK counting in mouse spleens, splenic cryosections were fixed in ice-cold acetone and then blocked in 5% BSA/PBS at RT for 1 h. APC-conjugated anti-mouse CD41 (10 μg/mL) was incubated at RT for 2 h. Cell nuclei were stained with 5 μg/mL DAPI. Samples were visualized with a Leica TCS SP8 confocal microscope.

### In vitro differentiation of MKs and platelet production analysis

Female WT and *Zyx*^−/−^ mice were sacrificed on day 14.5 of pregnancy. Fetal livers were harvested and single-cell suspension was prepared as previously described [45]. For isolation of HPCs from mouse BM cells, the femora and tibia of 6–8 weeks old mice were isolated and the BM was flushed by EasySep buffer and homogenized as previously described [45]. FL and BM HPCs were isolated by a mouse HPC isolation kit based on the manufacturer’s protocol. The isolated HPCs (3 × 10^5^/mL) were cultured in Stemspan^TM^ SFEM in the presence of 1% penicillin, 1% streptomycin, and 20 ng/mL recombinant murine TPO at 37 °C for 6 days.

The cell suspension was collected from day 3 to 6 and centrifuged at 300 × g for 5 min to separate culture-derived MKs (pellet) and platelet-sized particles (supernatant). The cell pellet was washed with PBS and resuspended in cell staining buffer. Non-specific binding was blocked by 20 μg/mL 2.4G2 at RT for 10 min. Then cells were labeled with APC-conjugated rat anti-mouse CD41 antibody (MWReg30, 2 μg/mL) and FITC-conjugated rat anti-mouse GPIbα antibody (Xia.G5, 1:10) at RT for 20 min. After being washed with PBS, cells were labeled with 5 μg/mL PI at RT for 10 min and then measured by flow cytometer. The percentage of CD41^+^ and GPIbα^+^ on cells was analyzed in the PI^−^ population. The supernatant was further centrifuged at 3,500 rpm for 2 min, and the pelleted platelet-sized particles were washed with CGS resuspended in cell staining buffer, and labeled with APC-conjugated rat anti-mouse CD41 antibody (MWReg30, 2 μg/mL) and FITC-conjugated rat anti-mouse GPIbα antibody (Xia.G5, 1:10) at RT for 20 min. After washed with CGS buffer, platelet-sized particles were stained with 5 μg/mL PI at RT for 10 min and then washed and measured by flow cytometer. The percentage of CD41^+^ and GPIbα^+^ on platelet-sized particles was analyzed in the PI^−^ population.

### Proteomic sample preparation

Washed platelets (1 × 10^8^) from WT and *Zyx*^−/−^ mice were lysed with 100 μL lysis buffer (0.05 M NH_4_HCO_3_, 2% sodium deoxycholate, 0.025 M NaCl). After centrifuged at 14,000 × g for 10 min to remove cell debris, protein concentration was measured by BCA kit and adjusted to the same level by lysis buffer. DTT (2 mM) was added to the platelet lysates and incubated at 95 °C for 3 min. Proteins were hydrolyzed by 2.5 μg trypsin (V528A, Promega, Madison, WI, USA) at 37 °C for 4 h and further hydrolyzed by the addition of 100 μL lysis buffer and 2.5 μg trypsin at 37 °C overnight. The reaction was stopped by 1% formic acid. After centrifuged at 14,000 × g at 4 °C for 15 min, supernatant was collected and lyophilized. The differential proteomic analysis was conducted by Human Phenome Institute (Fudan University, Shanghai).

### RNA extraction and qRT-PCR

Total RNA was extracted from washed platelet from 4–8 mice or 1 × 10^5^ Dami cells (ATCC, Manassas, VA, USA) using TRIzol reagent (15596026, Thermo Fisher Scientific), and the RNA concentration was determined using the NanoDrop-2000 Spectrophotometer (Thermo Fisher Scientific). Then, the RNA was reversely transcribed to cDNA using the RevertAid First Strand cDNA synthesis kit (k1622, Thermo Fisher Scientific) according to the manufacturer’s instruction. qRT-PCR was performed using primers specific for GPIbα, GPIbβ, GPIX, and GAPDH on a LightCycler 96 instrument (Roche, Indianapolis, IN, USA). Primer sequences were as follows: GPIbα-mus forward, 5'-CCAACAGAACAATTGCGTGAG-3'; GPIbα-mus reverse, 5'-GAGGAGGGTCCCAAAGAAGC-3'; GPIbβ-mus forward, 5'-CTACGTGGCGGAGGATGAG-3'; GPIbβ-mus reverse, 5'-GCCAGGTGAAGGTGAGGGTC-3'; GPIX-mus forward, 5'-TGCCAGTCCTTGGAAACCG-3'; GPIX-mus reverse, 5'-TCGCACTGAACGCAGGCTAT-3'; GAPDH-mus forward, 5'-AGCAGGCATCTGAGGGCCCA-3'; GAPDH-mus reverse, 5'-GAGAGCAATGCCAGCCCCGG-3'; GPIbα-homo forward, 5'-CCTTCGGAGGTCTTTCTGCT-3'; GPIbα-homo reverse, 5'-TCGGCTGAGTGAGCGAGTGT-3'; GPIbβ-homo forward, 5'-TGATGATGCTGCTGCTGTGC-3'; GPIbβ-homo reverse, 5'-CTCCCAAGCCTGGAATAGTGC-3'; GPIX-homo forward, 5'-AAAGCCTACCATCCACATTGC-3'; GPIX-homo reverse, 5'-CTCCATCCAGAGGGAAGCAG-3'; GAPDH-homo forward, 5'-GGACCTGACCTGCCGTCTAG-3'; GAPDH-homo reverse, 5'-GTAGCCCAGGATGCCCTTGA-3'. Each reaction contained 4 μL cDNA, 6 μL SYBR Green (04913914001, Roche) and 3 μL each of forward and reverse primers (10 μM). Examination of the melting curve for non-specific peaks was performed to ensure the specificity of PCR reactions, and mRNA levels were determined from Ct-values.

### RNA interference

siRNA targeting zyxin and negative control siRNA were purchased from GenePharma (Shanghai, China). Negative control siRNA (sense, 5'-UUCUCCGAACGUGUCACGUTT-3'; antisense, 5'-ACGUGACACGUUCGGAGAATT-3') or zyxin siRNAs (si*ZYX*-1, sense, 5'-GCCUCAGGUCCAACUCCAUTT-3'; antisense, 5'-AUGGAGUUGGACCUGAGGCTT-3'; si*ZYX*-2, sense, 5'-GGAUCUGGGUCACAACCAATT-3'; antisense, 5'-UUGGUUGUGACCCAGAUCCTT-3') were transfected into Dami cells by Lipofectamine™ 2000 Transfection Reagent according to the manufacturer’s instruction. Approximately 5 × 10^5^ cells were transfected with 175 pmol of siRNA. Briefly, 4 μL Lipofectamine™ 2000 Transfection Reagent and 8 μL siRNA (20 μM) were diluted with 200 μL reduced serum Opti-MEM I Medium (31985-062, Gibco, Thermo Fisher Scientific), respectively. Then, the complex was mixed and added to the cells and another 1 mL RPMI-1640 medium supplemented with 10% FBS was added to the cells. The cells were cultured for 48 h after transfection and then harvested. To verify the effect of siRNA transfection, qRT-PCR for zyxin gene expression and Western blot for zyxin protein expression were performed.

### Flow cytometry

For detecting platelet GPIb-IX surface level, platelets in PRP from WT and *Zyx*^−/−^ mice were labeled by FITC-conjugated anti-mouse GPIbα, GPIbβ, and GPIX antibodies (1:5) at RT for 30 min. For detecting reticulated platelets, platelets in whole blood were labeled by thiazole orange (0.5 μg/mL) and PE-conjugated anti-mouse CD41 antibody (5 μg/mL). For detecting Dami GPIb-IX surface level, cells ( < 1 × 10^6^) were collected and washed with PBS. Dami cells were then resuspended in 0.5% BSA/PBS and incubated with 10 μg/mL anti-GPIbα (6F3') and anti-GPIX (FMC25) antibodies at RT for 1 h. After being washed with 0.5% BSA/PBS twice, cells were stained with FITC-conjugated goat anti-mouse IgG (1 μg/mL) at RT for 1 h and then washed twice with 0.5% BSA/PBS. Platelets and Dami cells were examined by flow cytometry.

### Western blot

For detection of mouse platelet proteins, washed WT mouse platelets (3 × 10^8^/mL) and *Zyx*^−/−^ platelets (1.5 × 10^8^/mL) were lysed with an equal volume of 2 × lysis buffer (100 mM Tris, pH 7.4, 2% Triton X-100, 20 mM MgCl_2_, 300 mM NaCl) containing phenylmethylsulfonyl fluoride (1 mM) and protease inhibitor cocktail on ice for 30 min. For detection of Dami cell proteins, approximately 2 × 10^6^ cells were lysed in 100 μL 1 × lysis buffer on ice for 30 min and the supernatant was separated from insoluble cell fractions by centrifugation at 12,000 × g for 10 min at 4 °C. Protein concentration was measured by the BCA protein assay kit and adjusted to the same level by lysis buffer. Proteins were separated by SDS-PAGE and immunoblotted with specific antibodies.

### Confocal microscopy analysis of platelets and MKs

For confocal analysis of resting platelets, washed mouse platelets (1 × 10^6^ in 100 μL Modified Tyrode’s buffer) were fixed with an equal volume of 4% PFA and spun to slides. For spread platelets, the slides were first coated with 30 μg/mL fibrinogen diluted in 0.1 M NaHCO_3_ (pH8.3) at 4 °C overnight and blocked with 5% BSA. Washed mouse platelets (1 × 10^**7**^/mL) were allowed to adhere and spread on the fibrinogen-coated surface in the presence of 0.1 U/mL thrombin for 2 h at 37 °C. Platelets were fixed with 4% PFA. For spread MKs, the confocal dishes were first coated with 100 μg/mL fibrinogen or 50 μg/mL type I collagen at 4 °C overnight and blocked with 5% BSA. MKs cultured from mouse FL HPCs were resuspended in DMEM medium and allowed to adhere and spread on a fibrinogen- or collagen-coated surface for 3 h at 37 °C with 5% CO_2_. MKs were fixed with 4% PFA. Platelets and MKs were then permeabilized with 0.1% Triton X-100 and blocked with 5% BSA at RT. Platelets and MKs were stained with specific primary antibodies (10 μg/mL) and Alexa Fluor 488- or 555-conjugated secondary antibodies (4 μg/mL). For F-actin staining, Alexa Fluor 488-conjugated phalloidin (0.165 μM) was incubated for 15 min. Sufficient washing was performed after each step. Platelets and MKs were observed with a LEICA TCS SP8 confocal microscope with a 63x oil immersion lens.

### Co-immunoprecipitation

Washed platelet (3 × 10^8^/mL) were lysed with an equal volume of 2 × lysis buffer containing phenylmethylsulfonyl fluoride (1 mM), protease inhibitor cocktail, NaF (2 mM), and Na_3_VO_4_ (2 mM) on ice for 30 min. After centrifugation at 15,000 × g at 4 °C for 4 min, the supernatants were immunoprecipitated with antibodies overnight. After incubation with Protein G Plus Agarose beads at 4 °C for 4 h, the beads were washed thoroughly and analyzed by immunoblotting.

### Construction of zyxin mutants and adenovirus infection

Adenoviruses expressing WT zyxin and zyxin mutants were purchased from Hanbio Technology Ltd (Shanghai, China). Briefly, cDNAs of WT zyxin and zyxin mutants zyx^43–564^ (lacking α-actinin/NMMHC-IIA binding site) and zyx^F71, 93, 105, 115A^ (zyx^4F > A^, lacking VASP binding site) were subcloned into adenovirus vector pAdEasy-EF1-MCS-CMV-GFP with a 5' three flag tag sequence. Plasmids were transfected into 293 cells to obtain viral stocks.

*Zyx*^−/−^ mouse FL HPCs were sorted and cultured in SFEM in the presence of 1% penicillin, 1% streptomycin, and 20 ng/mL TPO at 37 °C in 24-well plates (2 × 10^5^/mL). The next day cells were infected with adenoviruses expressing WT zyxin; zyxin mutants zyx^43–564^, zyx^F71, 93, 105, 115A^, and vehicle vector at a multiplicity of infection of 600. Cell suspensions were collected on day 4 and 5 (the day for HPC plating was day 0) and culture-derived MKs and platelet-sized particles were isolated as described above. The percentage of GFP^+^/CD41^+^ and GFP^+^/GPIbα^+^ double-positive cells and platelet-sized particles were analyzed by flow cytometry and adjusted based on the infection efficiency. For Western blot analysis of WT zyxin and zyxin mutant expression, cells were collected on day 3. Approximately 3 × 10^5^ cells were lysed in 30 μL 1 × lysis buffer on ice for 30 min, and the supernatant was separated from insoluble cell fractions by centrifugation at 12,000 × g for 10 min at 4 °C. Protein concentration was measured by the BCA protein assay kit and adjusted to the same level by lysis buffer. Proteins were separated by SDS-PAGE and immunoblotted with specific antibodies.

### Statistical analysis

Statistical analysis was performed using GraphPad Prism 8 software. Shapiro–Wilk test and Brown–Forsythe test was done for normality and variance, respectively. Numeric data were analyzed using one-way (for a single variant) or two-way (for multiple variants) ANOVA. Two groups were compared by the two-tailed unpaired Student’s *t*-test or Mann–Whitney test (when Gaussian distribution was not assumed). Different levels of significance are indicated as *P < 0.05, **P < 0.01, and ***P < 0.001. All animal experiments were subject to randomization based on litter. No animals or samples were excluded from the study. The sample size was predetermined based on the variability observed in prior experiments and on preliminary data. Investigators were not blinded to outcome assessment.

## Supplementary information

Supplementary information-final

## Data Availability

The data that support the findings of this study are available from the corresponding author upon reasonable request.

## References

[1] Machlus KR, Italiano JE (2013). The incredible journey: from megakaryocyte development to platelet formation. J Cell Biol.

[2] Eckly A, Heijnen H, Pertuy F, Geerts W, Proamer F, Rinckel JY (2014). Biogenesis of the demarcation membrane system (DMS) in megakaryocytes. Blood.

[3] Avecilla ST, Hattori K, Heissig B, Tejada R, Liao F, Shido K (2004). Chemokine-mediated interaction of hematopoietic progenitors with the bone marrow vascular niche is required for thrombopoiesis. Nat Med.

[4] Pitchford SC, Lodie T, Rankin SM (2012). VEGFR1 stimulates a CXCR4-dependent translocation of megakaryocytes to the vascular niche, enhancing platelet production in mice. Blood.

[5] Ghalloussi D, Dhenge A, Bergmeier W (2019). New insights into cytoskeletal remodeling during platelet production. J Thromb Haemost.

[6] Antkowiak A, Viaud J, Severin S, Zanoun M, Ceccato L, Chicanne G (2016). Cdc42-dependent F-actin dynamics drive structuration of the demarcation membrane system in megakaryocytes. J Thromb Haemost.

[7] Italiano JE, Lecine P, Shivdasani RA, Hartwig JH (1999). Blood platelets are assembled principally at the ends of proplatelet processes produced by differentiated megakaryocytes. J Cell Biol.

[8] Kunishima S, Saito H (2010). Advances in the understanding of MYH9 disorders. Curr Opin Hematol.

[9] Kunishima S, Okuno Y, Yoshida K, Shiraishi Y, Sanada M, Muramatsu H (2013). ACTN1 mutations cause congenital macrothrombocytopenia. Am J Hum Genet.

[10] Nurden P, Debili N, Coupry I, Bryckaert M, Youlyouz-Marfak I, Solé G (2011). Thrombocytopenia resulting from mutations in filamin A can be expressed as an isolated syndrome. Blood.

[11] Pleines I, Woods J, Chappaz S, Kew V, Foad N, Ballester-Beltrá J (2017). Mutations in tropomyosin 4 underlie a rare form of human macrothrombocytopenia. J Clin Invest.

[12] Stritt S, Nurden P, Turro E, Greene D, Jansen SB, Westbury SK (2016). A gain-of-function variant in DIAPH1 causes dominant macrothrombocytopenia and hearing loss. Blood.

[13] Sui Z, Nowak RB, Sanada C, Halene S, Krause DS, Fowler VM (2015). Regulation of actin polymerization by tropomodulin-3 controls megakaryocyte actin organization and platelet biogenesis. Blood.

[14] Hoffman LM, Nix DA, Benson B, Boot-Hanford R, Gustafsson E, Jamora C (2003). Targeted disruption of the murine zyxin gene. Mol Cell Biol.

[15] Smith MA, Hoffman LM, Beckerle MC (2014). LIM proteins in actin cytoskeleton mechanoresponse. Trends Cell Biol.

[16] Hoffman LM, Jensen CC, Kloeker S, Wang CL, Yoshigi M, Beckerle MC (2006). Genetic ablation of zyxin causes Mena/VASP mislocalization, increased motility, and deficits in actin remodeling. J Cell Biol.

[17] Smith MA, Blankman E, Gardel ML, Luettjohann L, Waterman CM, Beckerle MC (2010). A zyxin-mediated mechanism for actin stress fiber maintenance and repair. Dev Cell.

[18] Hoffman LM, Jensen CC, Chaturvedi A, Yoshigi M, Beckerle MC (2012). Stretch-induced actin remodeling requires targeting of zyxin to stress fibers and recruitment of actin regulators. Mol Biol Cell.

[19] Li B, Trueb B (2001). Analysis of the alpha-actinin/zyxin interaction. J Biol Chem.

[20] Drees B, Friederich E, Fradelizi J, Louvard D, Beckerle MC, Golsteyn RM (2000). Characterization of the interaction between zyxin and members of the Ena/vasodilator-stimulated phosphoprotein family of proteins. J Biol Chem.

[21] Li P, Wei G, Cao Y, Deng Q, Han X, Huang X (2018). Myosin IIa is critical for cAMP-mediated endothelial secretion of von Willebrand factor. Blood.

[22] Dcraene C, Garçon L, Lacout C, Sabri S, Auffray C, Vainchenker W (2004). Zyxin is up-regulated during megakaryocytic differentiation of human UT-7/c-mpl cells. Biochem Biophys Res Commun.

[23] Macalma T, Otte J, hensler ME, Bockholt SM, Louis HA, Kalff-Suske M (1996). Molecular characterization of human zyxin. J Biol Chem.

[24] Kile BT, Panopoulos AD, Stirzaker RA, Hacking DF, Tahtamouni LH, Willson TA (2007). Mutations in the cofilin partner Aip1/Wdr1 cause autoinflammatory disease and macrothrombocytopenia. Blood.

[25] Pleines I, Dütting S, Cherpokova D, Eckly A, Meyer I, Morowski M (2013). Defective tubulin organization and proplatelet formation in murine megakaryocytes lacking Rac1 and Cdc42. Blood.

[26] Kunishima S, Kobayashi R, Itoh TJ, Hamaguchi M, Saito H (2009). Mutation of the beta1-tubulin gene associated with congenital macrothrombocytopenia affecting microtubule assembly. Blood.

[27] Savoia A, Kunishima S, De Rocco D, Zieger B, Rand ML, Pujol-Moix N (2014). Spectrum of the mutations in Bernard–Soulier syndrome. Hum Mutat.

[28] Bury L, Falcinelli E, Chiasserini D, Springer TA, Italiano JE, Gresele P (2016). Cytoskeletal perturbation leads to platelet dysfunction and thrombocytopenia in variant forms of Glanzmann thrombasthenia. Haematologica.

[29] Estevez B, Kim K, Delaney MK, Stojanovic-Terpo A, Shen B, Ruan C (2016). Signaling-mediated cooperativity between glycoprotein Ib-IX and protease-activated receptors in thrombin-induced platelet activation. Blood.

[30] Dong JF, Gao S, López JA (1998). Synthesis, assembly, and intracellular transport of the platelet glycoprotein Ib-IX-V complex. J Biol Chem.

[31] López JA, Leung B, Reynolds CC, Li CQ, Fox JE (1992). Efficient plasma membrane expression of a functional platelet glycoprotein Ib-IX complex requires the presence of its three subunits. J Biol Chem.

[32] Greenberg SM, Rosenthal DS, Greeley TA, Tantravahi R, Handin RI (1988). Characterization of a new megakaryocytic cell line: the Dami cell. Blood.

[33] Strassel C, Eckly A, Léon C, Petitjean C, Freund M, Cazenave J (2009). Intrinsic impaired proplatelet formation and microtubule coil assembly of megakaryocytes in a mouse model of Bernard–Soulier syndrome. Haematologica.

[34] Meyer SC, Sanan DA, Fox JE (1998). Role of actin-binding protein in insertion of adhesion receptors into the membrane. J Biol Chem.

[35] Pecci A, Ma X, Savoia A, Adelstein RS (2018). MYH9: structure, functions and role of non-muscle myosin IIA in human disease. Gene.

[36] Noris P, Pecci A (2017). Hereditary thrombocytopenias: a growing list of disorders. Hematol Am Soc Hematol Educ Program.

[37] Bachmann C, Fischer L, Walter U, Reinhard M (1999). The EVH2 domain of the vasodilator-stimulated phosphoprotein mediates tetramerization, F-actin binding, and actin bundle formation. J Biol Chem.

[38] Fradelizi J, Noireaux V, Plastino J, Menichi B, Louvard D, Sykes C (2001). ActA and human zyxin harbour Arp2/3-independent actin-polymerization activity. Nat Cell Biol.

[39] Lanzetti L (2007). Actin in membrane trafficking. Curr Opin Cell Biol.

[40] Jurak Begonja A, Hoffmeister KM, Hartwig JH, Falet H (2011). FlnA-null megakaryocytes prematurely release large and fragile platelets that circulate poorly. Blood.

[41] White JG, Sauk JJ (1984). The organization of microtubules and microtubule coils in giant platelet disorders. Am J Pathol.

[42] Partynska A, Gomulkiewicz A, Dziegiel P, Podhorska-Okolow M (2020). The role of zyxin in carcinogenesis. Anticancer Res.

[43] Rosner SR, Pascoe CD, Blankman E, Jensen CC, Krishnan R, James AL (2017). The actin regulator zyxin reinforces airway smooth muscle and accumulates in airways of fatal asthmatics. PLoS One.

[44] Han X, Li P, Yang Z, Huang X, Wei G, Sun Y (2017). Zyxin regulates endothelial von Willebrand factor secretion by reorganizing actin filaments around exocytic granules. Nat Commun.

[45] Schulze H (2016). Culture, expansion, and differentiation of murine megakaryocytes from fetal liver, bone marrow, and spleen. Curr Protoc Immunol.

