# Supplementary material for: Essential role of zyxin in platelet biogenesis and glycoprotein Ib-IX surface expression
Source: Cell Death Dis. 2021 Oct 16;12(11):955. doi: 10.1038/s41419-021-04246-x (PMC8520529; doi:10.1038/s41419-021-04246-x)
Supplement: Supplementary file 1 — Supplementary information-final [file 41419_2021_4246_MOESM1_ESM.docx]

**Supplementary figures and figure legends**

**
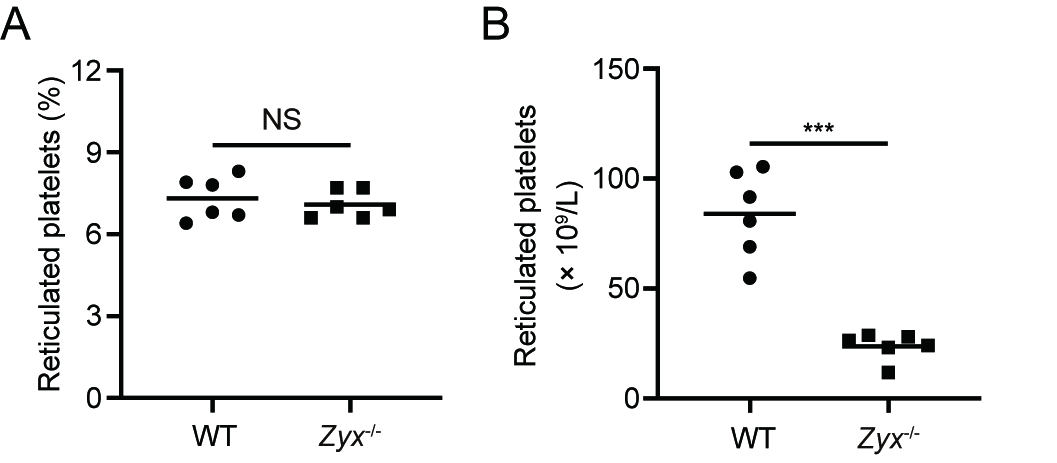
**

**Supplementary Fig.1 Reticulated platelets in *Zyx*^-/-^ mice. A** The percentage of reticulated platelets labeled with anti-CD41 antibody and thiazole orange in WT and *Zyx*^-/-^ mice was analyzed by flow cytometry. **B** The number of reticulated platelets in WT and *Zyx*^-/-^ was calculated by multiplying the platelet count by the percentage of reticulated platelets. n = 6 mice per genotype. Means are indicated by horizontal lines. ****P* < 0.001, compared with WT mice by unpaired Student’s *t* test with Welch correction. NS, not significant.


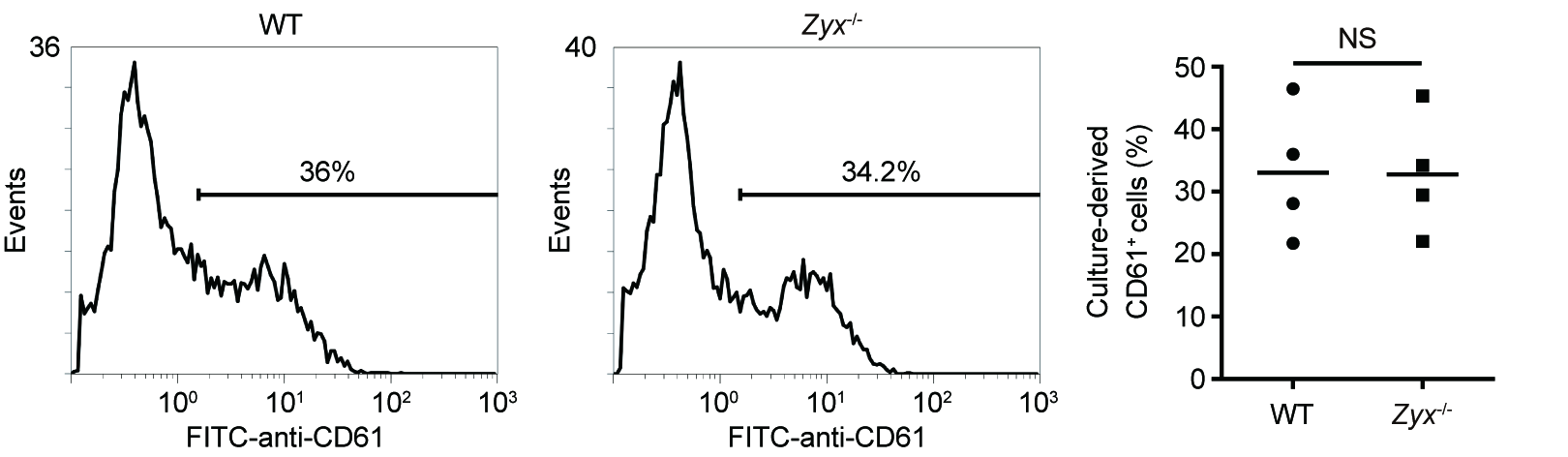


**Supplementary Fig.2 The percentage of CD61^+^ cells derived from *Zyx*^-/-^ FL HPCs.** WT and *Zyx*^-/-^ mouse FL HPCs were isolated and cultured. The CD61^+^ cells were analyzed on day 4 by flow cytometry. Data are from four independent experiments. Means are indicated by horizontal lines in the right panel. NS, not significant.


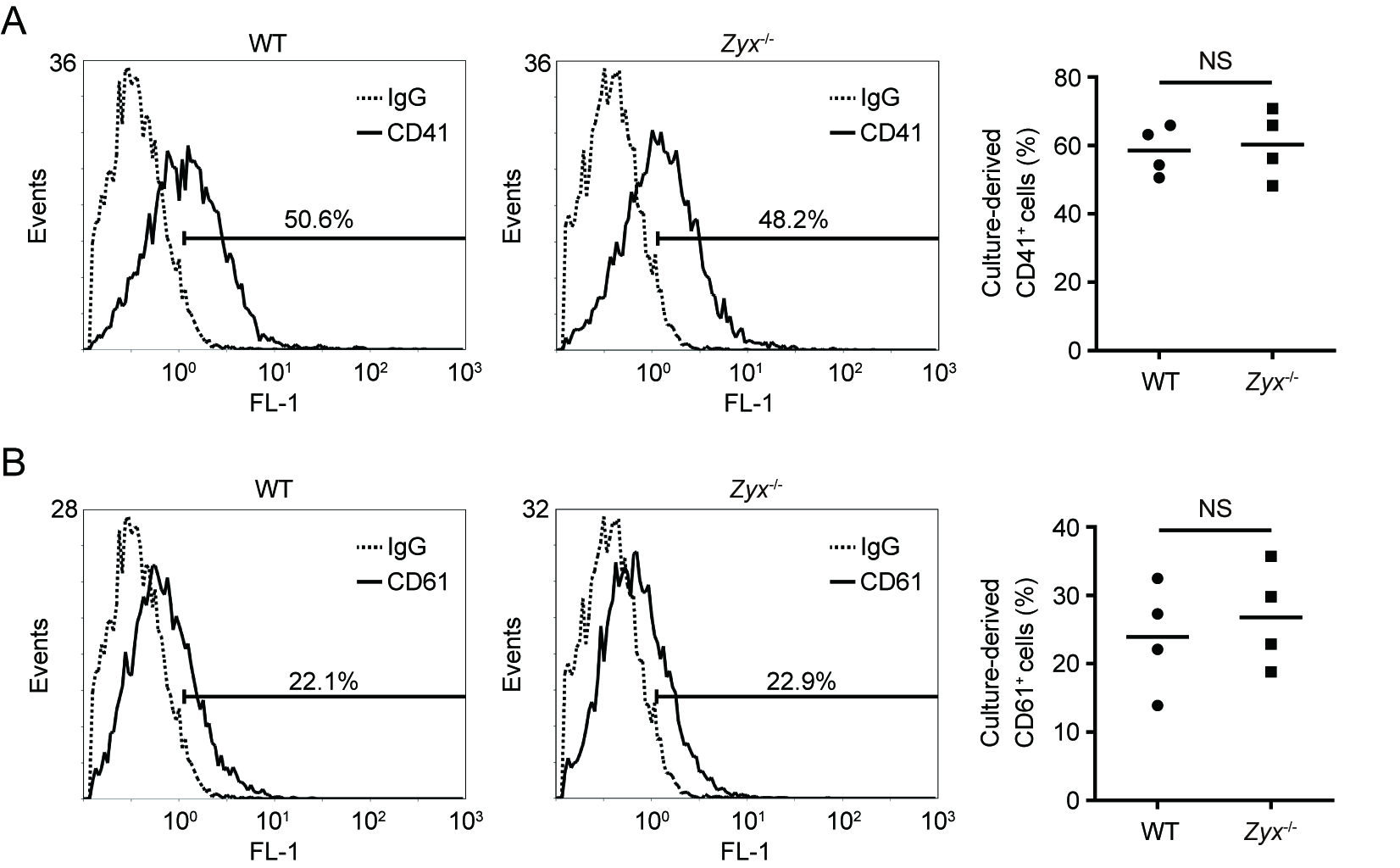


**Supplementary Fig.3 The percentage of CD41^+^ and CD61^+^ cells derived from *Zyx*^-/-^ BM HPCs.** WT and *Zyx*^-/-^ mouse BM HPCs cells were isolated and cultured. The percentages of CD41^+^ (**A**) and CD61^+^ (**B**) cells were analyzed on day 6 by flow cytometry. Data are from four independent experiments. Means are indicated by horizontal lines in the right panels. NS, not significant.

**
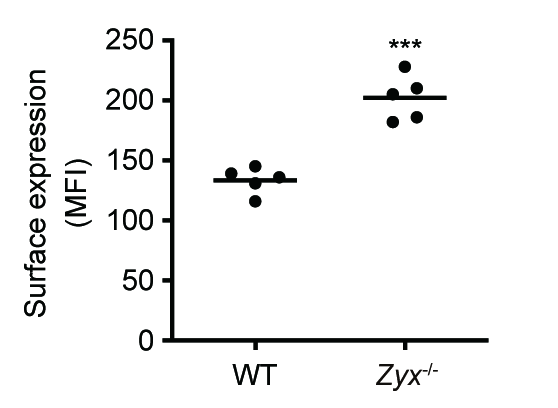
**

**Supplementary Fig. 4 Surface expression of GPIIb on *Zyx*^-/-^ platelets.** Surface level of GPIIb on WT and *Zyx*^-/-^ platelets were analyzed by flow cytometry; n = 5 mice per genotype. MFI, mean fluorescence intensity. Means are indicated by horizontal lines. ****P* < 0.001, compared with WT mice by unpaired Student’s *t* test.

**
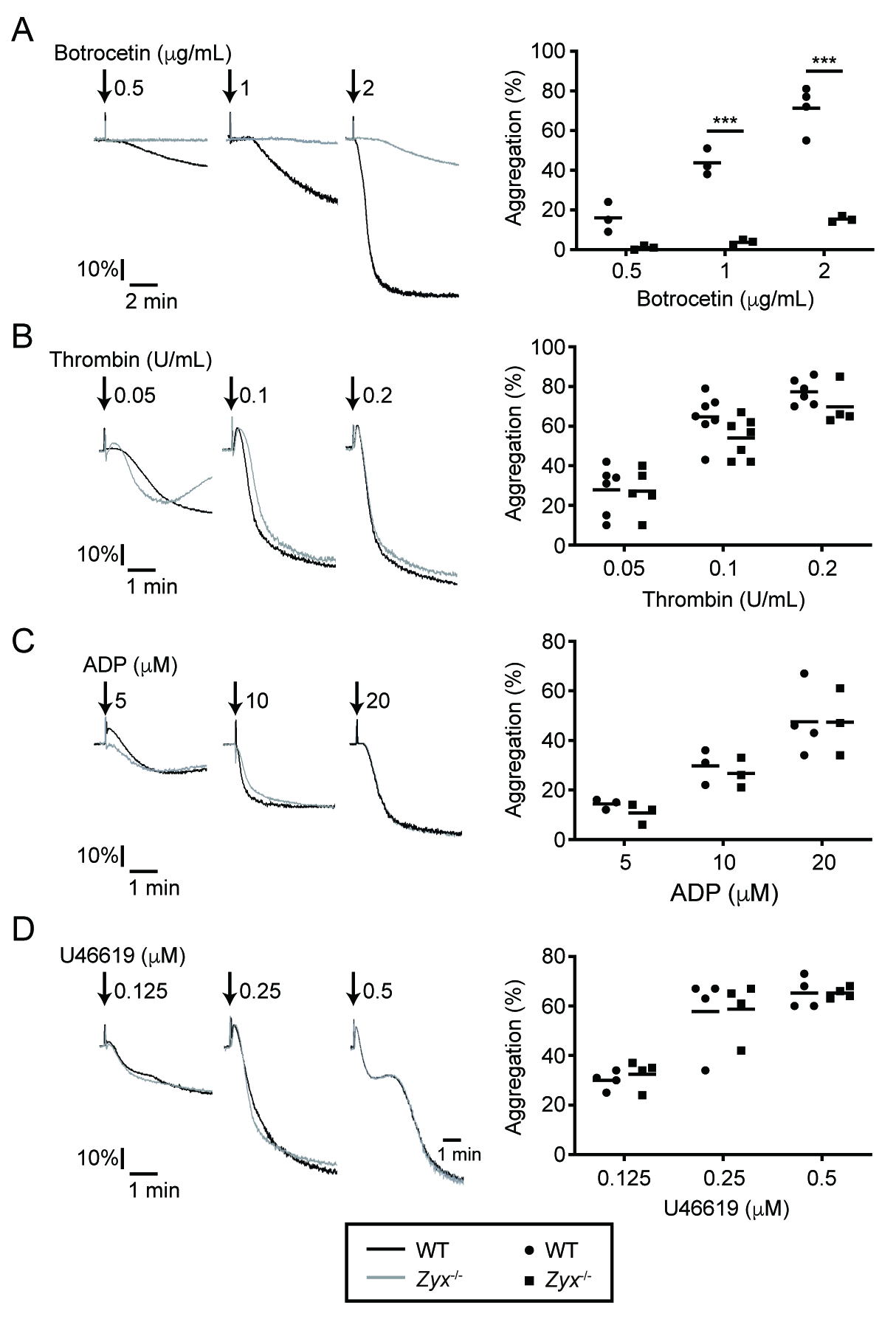
**

**Supplementary Fig. 5 Platelet aggregation in the absence of zyxin.** Platelet aggregation was induced by botrocetin (**A**), ADP (**C**), and U46619 (**D**) in platelet-rich plasma (PRP) and thrombin (**B**) in washed platelets from WT and *Zyx*^-/-^ mice. Left panels, the representative aggregation traces. Right panels, the quantification of maximal platelet aggregation rate. Data are from three (**A** and **C**), six (**B**) and four (**D**) independent experiments. Means are indicated by horizontal lines. ****P* < 0.001 compared with WT mice by two-way ANOVA followed by Bonferroni’s post hoc test.


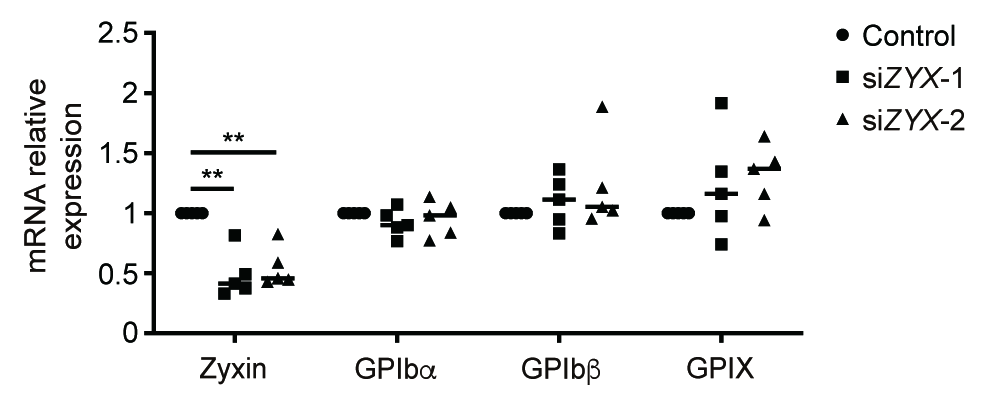


**Supplementary Fig. 6** **mRNA levels of zyxin, GPIbα, GPIbβ, and GPIX after zyxin knockdown.** Dami cells were transfected with siRNAs targeting zyxin (si*ZYX*-1 and -2) and negative control siRNA (control). Total RNA was extracted and the expression of zyxin, GPIbα, GPIbβ, and GPIX mRNA was measured by qRT-PCR. Expression was determined by a ratio relative to the internal control GAPDH and the data were expressed as the ratio relative to controls. Data are from five independent experiments. Means are indicated by horizontal lines. ***P* < 0.01, compared with control by Student’s *t* test.


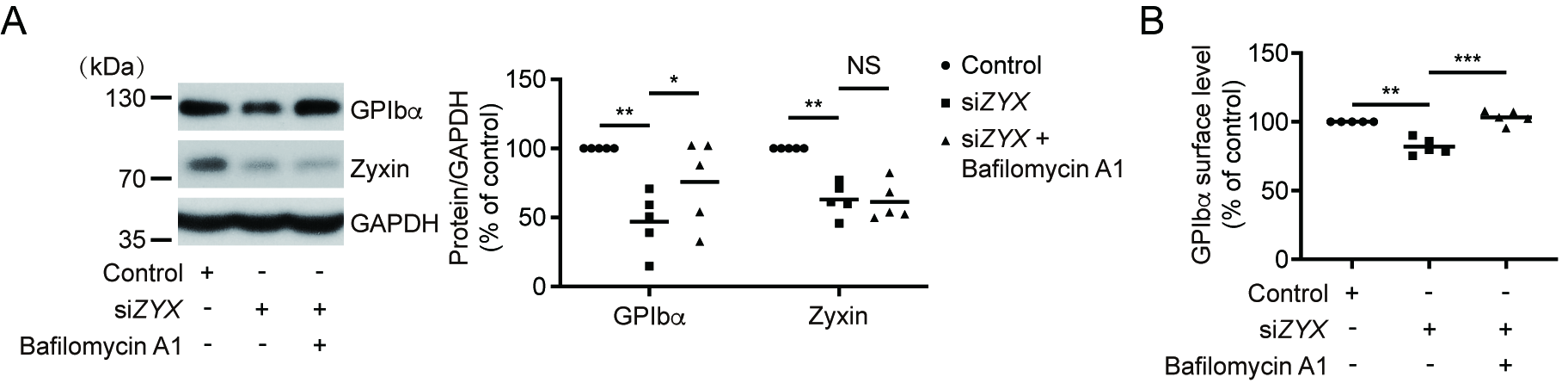


**Supplementary Fig. 7 Effect of bafilomycin A1 on zyxin knockdown-induced GPIbα reduction.** Dami cells were transfected with control siRNA (control) or zyxin siRNA (si*ZYX*). Bafilomycin A1 (100 nM) was added 12 h after zyxin siRNA transfection. **A** The total levels of GPIbα and zyxin were analyzed with Western blot. Blots are representative of five independent experiments. **B** The surface level of GPIbα was analyzed by flow cytometry. Data are from five independent experiments. Means are indicated by horizontal lines. **P* < 0.05, ***P* < 0.01, ****P* < 0.001, by one-way ANOVA followed by Dunnett’s post hoc test. NS, not significant.

**
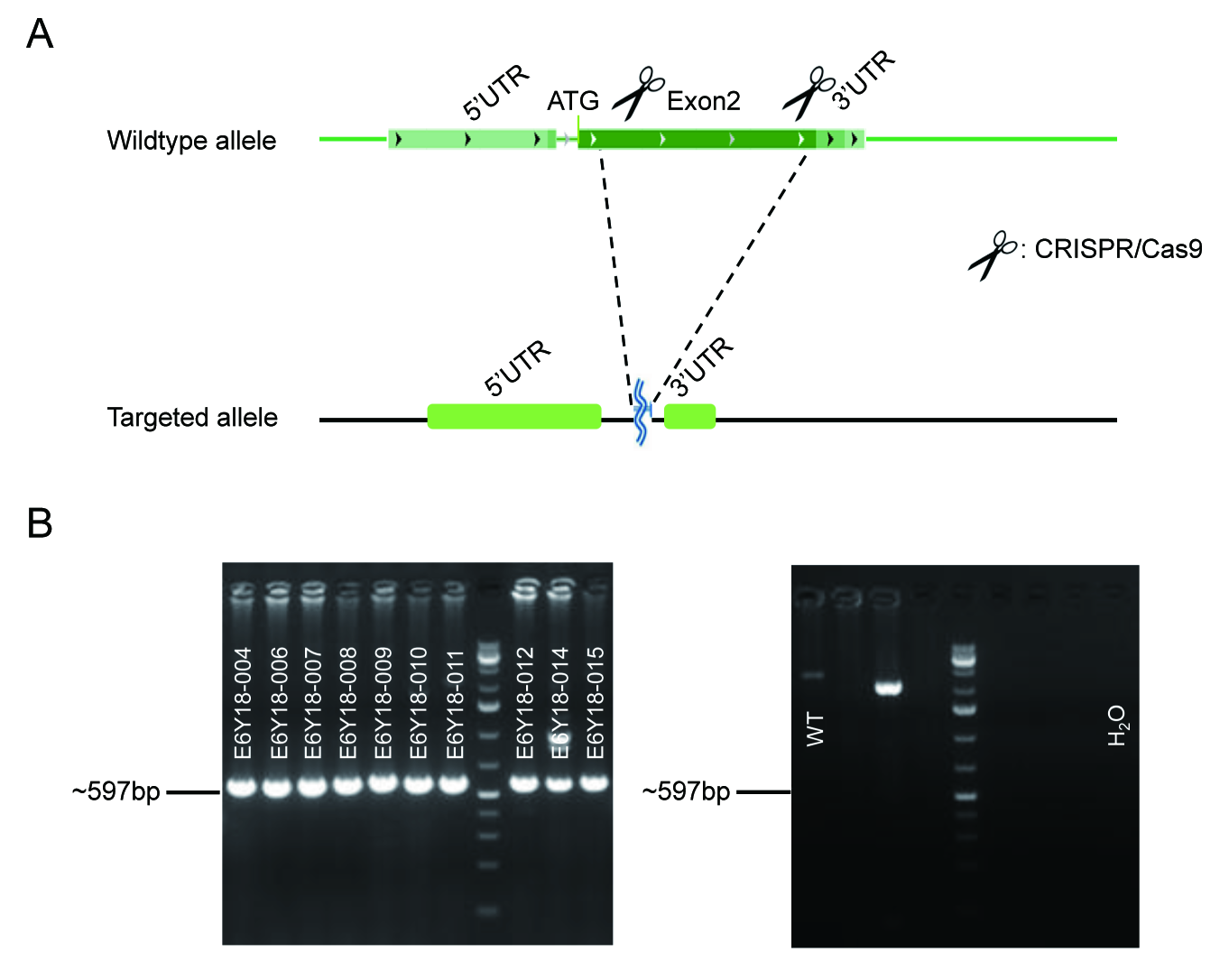
**

**Supplementary Fig. 8 Construction and verification of *Gp1ba^-/-^* mice.** **A** Schematic overview of the strategy to generate the *Gp1ba^-/-^* allele. Exon 2 of the mouse *Gp1ba* gene was targeted with two CRISPR gRNAs. The sequences of gRNA1: 5’-TCTCACAGTTTACTTCCAGC -3’; gRNA2: 5’- TATTGGGCACAGTGGGCATT -3’. **B** Identification of genotyping by PCR with the *Gp1ba*-Mut-F/*Gp1ba*-WT-R primers. Targeted region sequences: F; 5′-AGAAGCTCTGTTCCTCCAAAGGAC -3′, R; 5′-GGTAGTAGTGACCATGTAGCCTGAC -3′). Product size, WT: 2689bp; Mut: ~597bp; M, DNA marker.

**
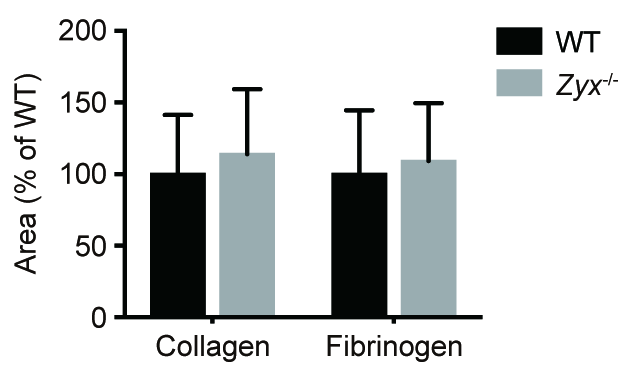
**

**Supplementary Fig. 9 Areas of spread MKs.** MKs cultured from mouse FL HPCs were allowed to spread on type I collagen and fibrinogen. Spreading areas of WT and *Zyx*^-/-^ MKs were analyzed by ImageJ software. No less than 60 MKs per genotype from seven (collagen) and eight (fibrinogen) independent experiments were analyzed. Data are expressed as means ± SD.

**
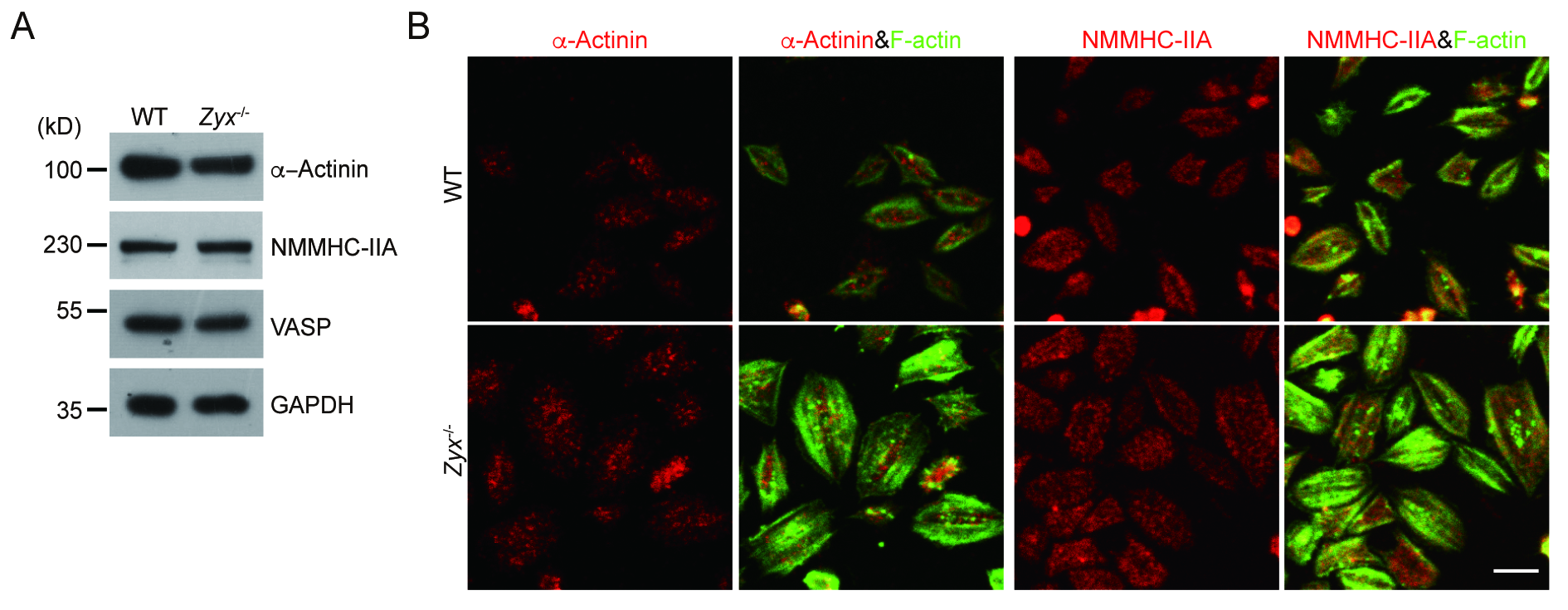
**

**Supplementary Fig. 10 α-Actinin or NMMHC-IIA distribution in *Zyx*^-/-^ platelets. A** Western blot analysis of α-actinin, NMMHC-IIA, and VASP in WT and *Zyx*^-/-^ platelets. Protein concentration has been adjusted to the same level between WT and *Zyx*^-/-^ platelet lysates. Blots are representative of five independent experiments. **B** Platelets were allowed to spread on fibrinogen in the presence of thrombin. Representative confocal images of spread WT and *Zyx*^-/-^ platelets stained for α-actinin or NMMHC-IIA (red), and F-actin (green). The original magnification of the images is ×630. Scale bar: 5 μm. Results are representative of at least five independent experiments.

**
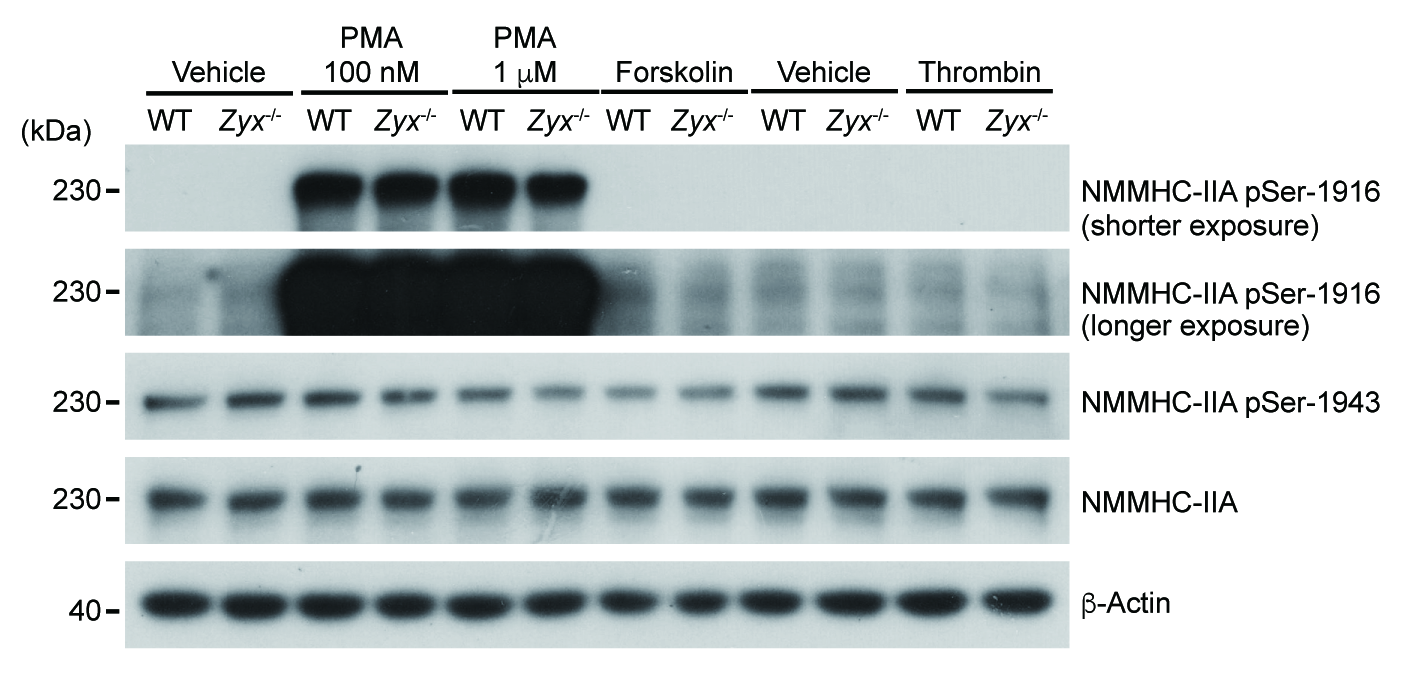
**

**Supplementary Fig. 11** **Zyxin deficiency does not affect NMMHC-IIA phosphorylation.** Washed platelets were treated with 100 nM PMA, 1 μM PMA, 10 μM forskolin, 0.05 U/mL thrombin, and vehicle control at RT for 15 min. Phosphorylation of NMMHC-IIA at Ser1916 and Ser1943 was analyzed by Western blot with anti-pSer-1916 and pSer-1943 antibodies. Protein concentration has been adjusted to the same level between WT and *Zyx*^-/-^ platelet lysates. Blots are representative of five independent experiments.

**
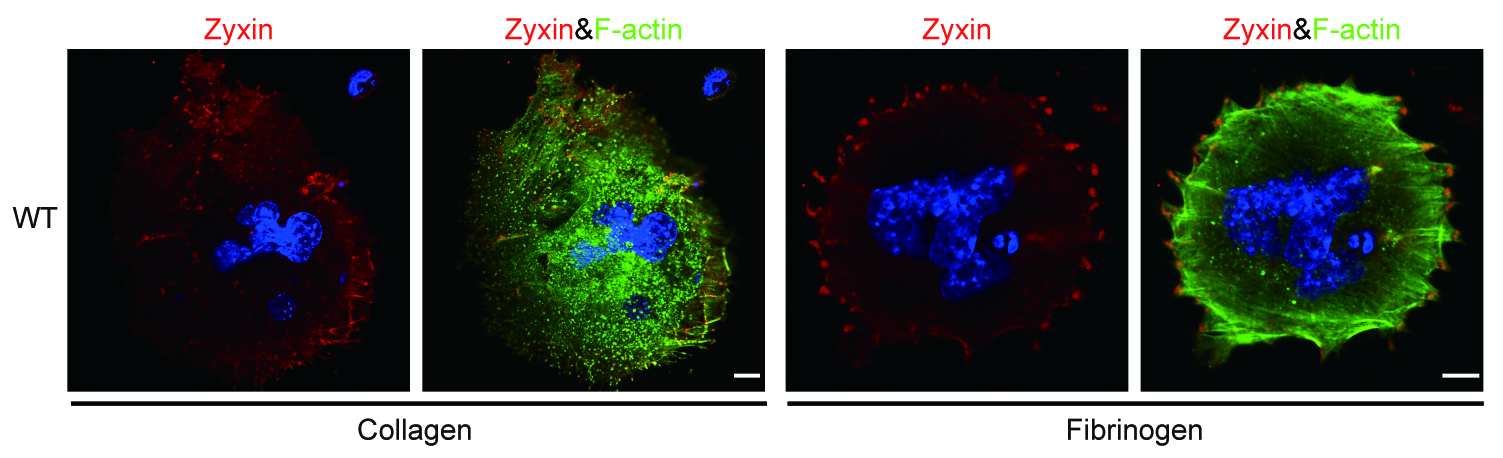
**

**Supplementary Fig. 12** **Zyxin localization in MKs.** MKs cultured from WT mouse FL HPCs were allowed to spread on type I collagen- or fibrinogen-immobilized surface. Zyxin was immunostained with the rabbit anti-zyxin antibody and 555-conjugated goat anti-rabbit IgG secondary antibody (red). F-actin was labeled with FITC-conjugated phalloidin (green). Nuclei were labeled with DAPI (blue). The original magnification of the images is ×630. Scale bar: 10 μm. Results are representative of five independent experiments.

**Supplementary tables**

**Supplementary table 1.** Hematologic analysis of *Zyx*^-/-^ mice.

| Hematological  parameter | WT  n = 21 | *Zyx^-/-^*  n = 17 |
| --- | --- | --- |
| WBC (10^9^/L) | 10.23 ± 2.65 | 10.01 ± 3.06 |
| NEUT (10^9^/L) | 1.36 ± 0.86 | 1.58 ± 1.46 |
| LYM (10^9^/L) | 8.58 ± 2.26 | 8.14 ± 2.05 |
| RBC (10^12^/L) | 10.18 ± 1.28 | 9.92 ± 1.55 |
| HGB (g/L) | 164.01 ± 19.13 | 164.42 ± 25.35 |
| MCV (fL) | 49.25 ± 1.34 | 50.81 ± 0.97 |

WBC, white blood cell; NEUT, neutrophil; LYM, lymphocyte; RBC, red blood cell; HGB, hemoglobin; MCV, mean corpuscular volume. Data are means ± SD.

**Supplementary table 2. Hematologic analysis of *Gp1ba^-/-^* mice.**

| Hematological  parameter | WT  n = 7 | *Gp1ba****^-/-^***  n = 5 |
| --- | --- | --- |
| PLT (10^9^/L) | 1134.31 ± 154.05 | 106.98 ± 28.26 ^***^ |
| MPV (fL) | 4.34 ± 0.17 | 11.48 ± 0.52 ^***^ |
| WBC (10^9^/L) | 10.14 ± 1.83 | 8.59 ± 2.21 |
| NEUT (10^9^/L) | 1.48 ± 0.44 | 2.11 ± 0.90 |
| LYM (10^9^/L) | 8.39 ± 1.47 | 5.85 ± 1.72 |
| RBC (10^12^/L) | 8.99 ± 0.50 | 7.32 ± 1.05 |
| HGB (g/L) | 120.43 ± 8.54 | 124.60 ± 2.90 |
| MCV (fL) | 45.78 ± 0.90 | 50.2 ± 4.72 |

PLT, platelet; MPV, mean platelet volume. Data are means ± SD; ^***^*P* < 0.001.
